# Using a Systems Approach to Explore the Mechanisms of Interaction Between Severe Covid-19 and Its Coronary Heart Disease Complications

**DOI:** 10.3389/fcvm.2022.737592

**Published:** 2022-02-16

**Authors:** Albertus A. Meyer, Edward H. Mathews, Andries G. S. Gous, Marc J. Mathews

**Affiliations:** ^1^Centre for Research in Continued Engineering Development (CRCED), North-West University, Potchefstroom, South Africa; ^2^Department of Physiology, Medical School, University of Pretoria, Pretoria, South Africa; ^3^Department of Industrial Engineering, Stellenbosch University, Stellenbosch, South Africa

**Keywords:** COVID-19, SARS-CoV-2, coronary heart disease, cardiovascular comorbidities, systems-approaches

## Abstract

*Frontiers* requested research on how a systems approach can explore the mechanisms of cardiovascular complications in Covid-19. The focus of this paper will thus be on these detailed mechanisms. It will elucidate the integrated pathogenic pathways based on an extensive review of literature. Many severe Covid-19 cases and deaths occur in patients with chronic cardiovascular comorbidities. To help understand all the mechanisms of this interaction, Covid-19 complications were integrated into a pre-existing systems-based coronary heart disease (CHD) model. Such a complete model could not be found in literature. A fully integrative view could be valuable in identifying new *pharmaceutical interventions*, help understand how *health factors* influence Covid-19 severity and give a fully integrated explanation for the Covid-19 *death spiral* phenomenon seen in some patients. Covid-19 data showed that CHD hallmarks namely, *Hypercoagulability, Hypercholesterolemia, Hyperglycemia/Hyperinsulinemia, Inflammation* and *Hypertension* have an important effect on disease severity. The pathogenic pathways that Covid-19 activate in CHD were integrated into the CHD model. This fully integrated model presents a visual explanation of the mechanism of interaction between CHD and Covid-19 complications. This includes a detailed integrated explanation of the death spiral as a result of interactions between *Inflammation*, endothelial cell injury, *Hypercoagulability* and hypoxia. Additionally, the model presents the aggravation of this *death spiral* through the other CHD hallmarks namely, *Hyperglycemia/Hyperinsulinemia, Hypercholesterolemia*, and/or *Hypertension*. The resulting model further suggests systematically how the pathogenesis of nine *health factors* (stress, exercise, smoking, etc.) and seven *pharmaceutical interventions* (statins, salicylates, thrombin inhibitors, etc.) may either aggravate or suppress Covid-19 severity. A strong association between CHD and Covid-19 for all the investigated *health factors* and *pharmaceutical interventions*, except for β-blockers, was found. It is further discussed how the proposed model can be extended in future to do computational analysis to help assess the risk of Covid-19 in cardiovascular disease. With insight gained from this study, recommendations are made for future research in potential new pharmacotherapeutics. These recommendations could also be beneficial for cardiovascular disease, which killed five times more people in the past year than Covid-19.

## Introduction

The coronavirus disease of 2019 (Covid-19) is caused by the infection of severe acute respiratory syndrome coronavirus 2 (SARS-CoV-2), which first emerged in December 2019 in Wuhan, China (1). In March 2020, the World Health Organization declared this disease a pandemic (2). As of 8 August 2021, the total number of confirmed global deaths were 4,285,421 (3).

It is widely accepted that Covid-19 severity is increased by respiratory complications such as hypoxia (4, 5). Critically ill patients developing hypoxia requires supplemental oxygen and/or mechanical ventilation (4, 5). Although this condition is respiratory related, this hypoxia is fueled by vascular complications which are documented in numerous autopsies (4, 6–9). Moreover, pre-existing cardiovascular related comorbidities are known risk factors that increase Covid-19 severity. These comorbidities include, among others, *Hypertension, Hyperglycemia/Hyperinsulinemia*, obesity and/or chronic cardiac disease (10–14).

Furthermore, hospitalized critically ill Covid-19 patients experience cardiovascular complications such as cardiac injury, thrombosis, arrhythmia, heart failure and myocardial dysfunction (15–19). This is again substantiated by autopsies that present various findings of vasculature damage that leads to a state of *Hypercoagulability* in deceased Covid-19 patients (4, 6–9).

Most severe Covid-19 patients also experience a chronic heightened *Inflammatory state*, especially within the alveoli and pulmonary capillaries (20–22). This may be as a result of the dysregulated hyperimmune response (20) and/or direct viral infection mediating inflammatory cell infiltration (11, 22).

Therefore, the prevailing viewpoints in literature are that most severe cases of Covid-19 (i) result in cardiovascular complications (4, 6–9) and/or (ii) are seen in patients with pre-existing cardiovascular comorbidities (10–14). A need therefore exists to further investigate the underlying mechanisms/pathogenesis between cardiovascular disease and Covid-19.

**Figure 1:**
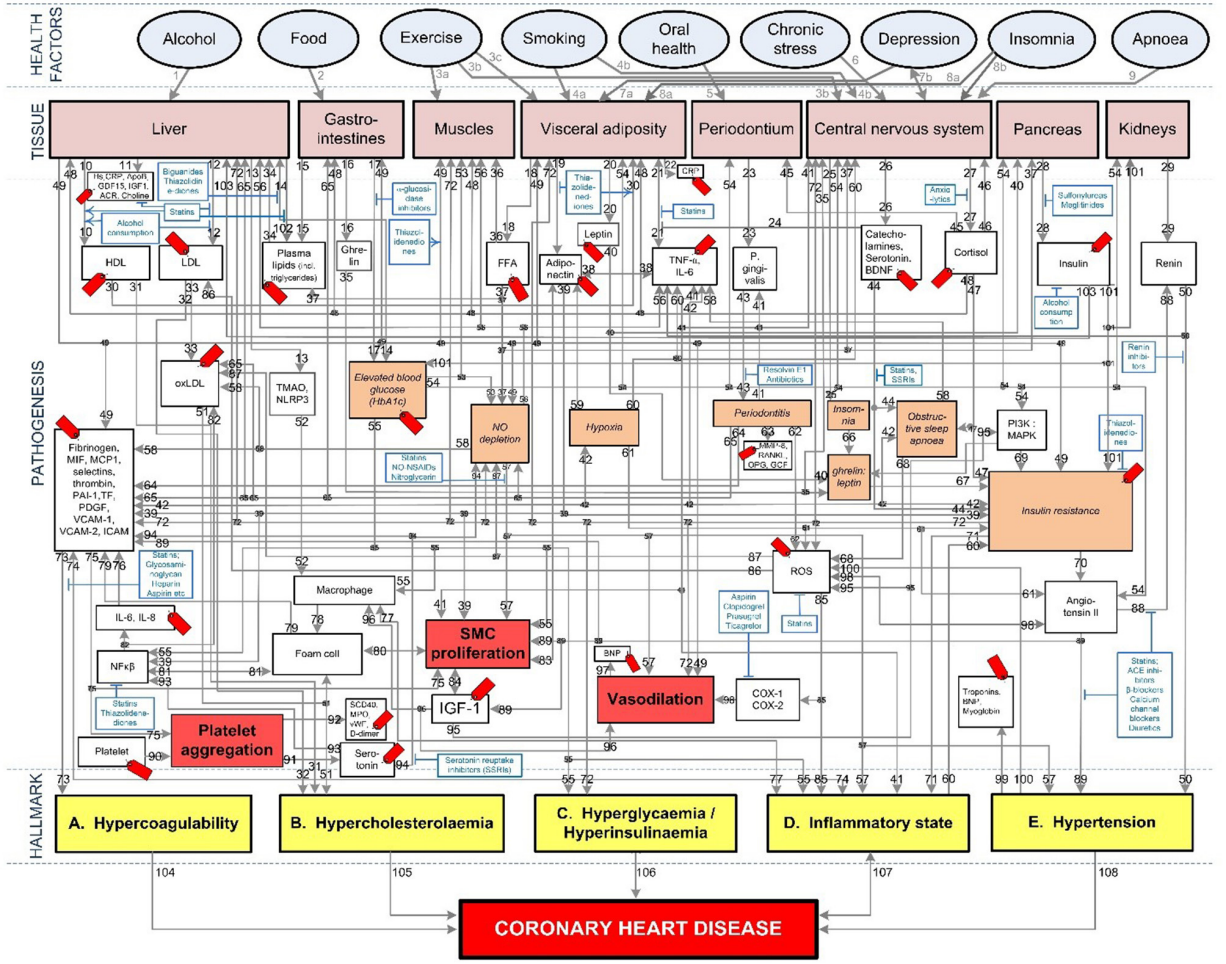
Existing model showing the mechanisms of coronary heart disease (23–28). The affective pathway of pharmaceuticals, blue boxes, is shown in Figure and salient serological biomarkers are indicated by the red tags (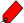). The blunted blue arrows denote antagonize or inhibit and pointed blue arrows denote up-regulate or facilitate. ACE, angiotensin-converting-enzyme; β-blocker, beta-adrenergic antagonists; BDNF, brain-derived neurotrophic factor; BNP, B-type natriuretic peptide; OX, cyclooxygenase; CRP, C-reactive protein; D-dimer, fibrin degradation product D; FFA, free fatty acids; GCF, gingival crevicular fluid; HbA1c, glycated hemoglobin A1c; HDL, high-density lipoprotein; Hs, homocysteine; ICAM, intracellular adhesion molecule; IGF-1, insulin-like growth factor-1; IL, interleukin; LDL, low-density lipoprotein; MAPK, mitogen-activated protein (MAP) kinase; MCP, monocyte chemoattractant protein; MIF, macrophage migration inhibitory factor; MMP, matrix metalloproteinase; MPO, myeloperoxidase; NFκβ, nuclear factor-κβ; NLRP3, Inflammasome responsible for activation of inflammatory processes as well as epithelial cell regeneration and microflora; NO, nitric oxide; NO-NSAIDs, combinational NO-non-steroidal anti-inflammatory drug; OPG, osteoprotegerin; oxLDL, oxidized LDL; PAI, plasminogen activator inhibitor; PDGF, platelet-derived growth factor; P. gingivalis, Porphyromonas gingivalis; PI3K, phosphatidylinositol 3-kinase; RANKL, receptor activator of nuclear factor kappa-beta ligand; ROS, reactive oxygen species; SCD-40, recombinant human sCD40 ligand; SMC, smooth muscle cell; SSRI, serotonin reuptake inhibitors; TF, tissue factor; TMAO, an oxidation product of trimethylamine (TMA); TNF-α, tumor necrosis factor-α; VCAM, vascular cell adhesion molecule; vWF, von Willebrand factor.

To fully investigate this, the pathogenesis of cardiovascular disease and Covid-19 needs to be integrated. Fortunately, most of the above mentioned vascular Covid-19 effects are included in an existing model of coronary heart disease (CHD) (Figure 1) (23, 24). These effects are depicted in Figure 1 as the following CHD hallmarks (yellow boxes): *(A) Hypercoagulability, (B) Hypercholesterolemia, (C) Hyperglycemia/Hyperinsulinemia, (D) Inflammatory state* and *(E) Hypertension*.

*Hypercholesterolemia* is a common CHD risk factor, known to aggravate vascular cell dysfunction, aggravate coagulation and upregulate inflammation (29–31). *Hypercholesterolemia (B)*, depicted in Figure 1, has only been partially linked to Covid-19 through high circulating cholesterol levels that may make a person more susceptible to infection (32). Although this might still be controversial, a recent molecular study showed that SARS-CoV-2 requires cholesterol for viral entry (33). Subsequently, another molecular study (yet unpublished) showed how cholesterol optimally positions furin for priming SARS-CoV-2 (34). In other words, cholesterol improves binding to ACE2 receptor, while producing a more infectious virion (34).

We envisage another association between increased Covid-19 severity and *Hypercholesterolemia*, through vascular complications that arise from high cholesterol levels. Since both *Hypercoagulability* and *Inflammation* are known risk factors for Covid-19 and *Hypercholesterolemia* influences both these hallmarks (23), we also included *Hypercholesterolemia* in our *integrated CHD/Covid-19 model* (more detailed discussions are given in sections Severe Covid-19 Patients With Existing Chronic Hypercholesterolemia and Effects of Different CHD *Pharmaceutical Interventions* on Covid-19 Severity).

All of the CHD Hallmarks identified in the CHD model (Figure 1) play a significant role in Covid-19 severity. The question is, will it be possible to use this CHD model and integrate the pathogenesis of Covid-19 with it?

In this paper we will attempt to integrate the CHD pathogenic pathways with those of severe Covid-19 complications, using a systems-based approach. This CHD/Covid-19 integration should provide insight into the following questions, some of which were requested by *Frontiers*:

1. Why do some patients with severe Covid-19 experience sudden death? (Section The Death Spiral: Inflammation, EC Injury, Coagulation, Vascular Leakage and Hypoxia)
2. How do CHD comorbidities influence this *death spiral*? (Section Covid-19 Aggravation in Patients With Pre-existing CHD Comorbidities)
3. How can an individual reduce the risk of developing severe Covid-19 from a cardiovascular point of view? (Sections Effects of Different *Health Factors* on Covid-19 Severity and Effects of Different CHD *Pharmaceutical Interventions* on Covid-19 Severity)
4. How can computational analysis help to assess the risk of COVID-19 in cardiovascular disease? (Section How Can Computational Analysis Help to Assess the Risk of Severity in Covid-19 in Cardiovascular Disease?)
5. Are there other opportunities in cardiovascular disease that can be derived from this paper and the Covid-19 crisis? (Section Are There Other Opportunities in Cardiovascular Disease That Can Be Derived From This Paper and the Covid-19 Crisis?).

We envisage that the proposed *integrated CHD/Covid-19 model* may help answer some of these questions, thereby potentially enhancing the future management of both Covid-19 and CHD.

## Method

**Figure 2:**
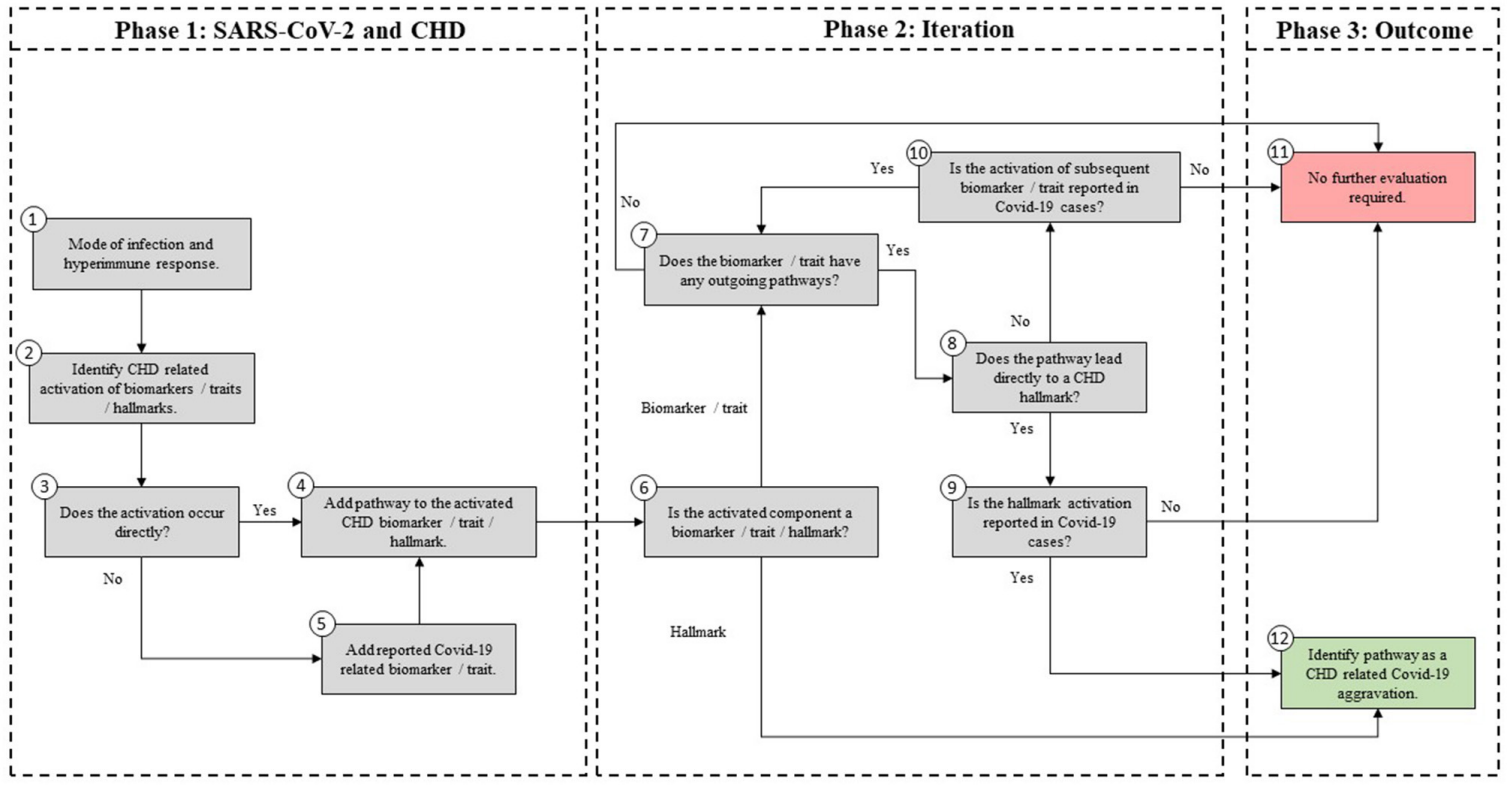
Methodology used to develop an Integrated Covid-19/CHD model.

**Figure 3:**
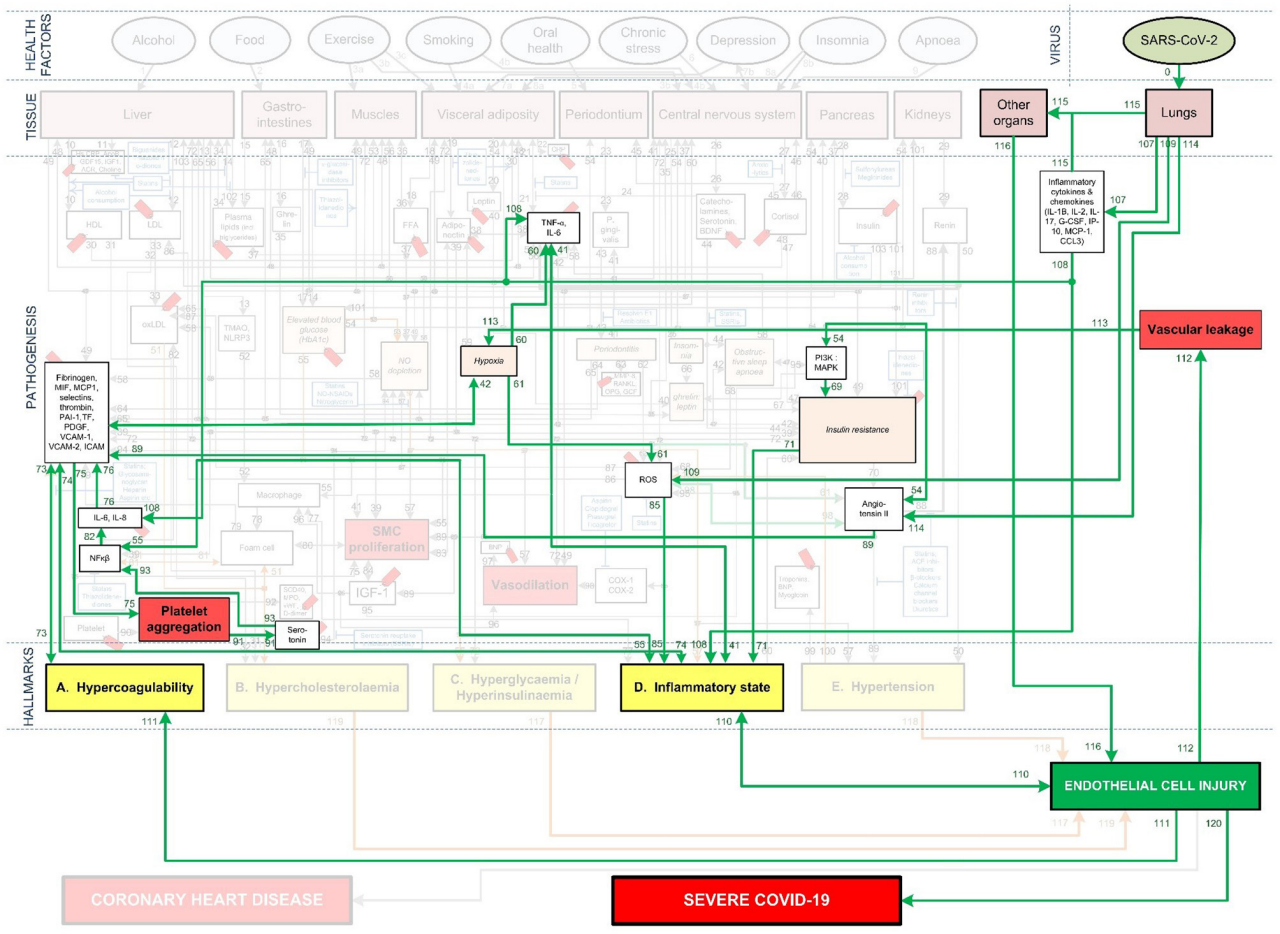
Integration of Covid-19 pathogenic pathways into the pathways of CHD.

**Figure 4:**
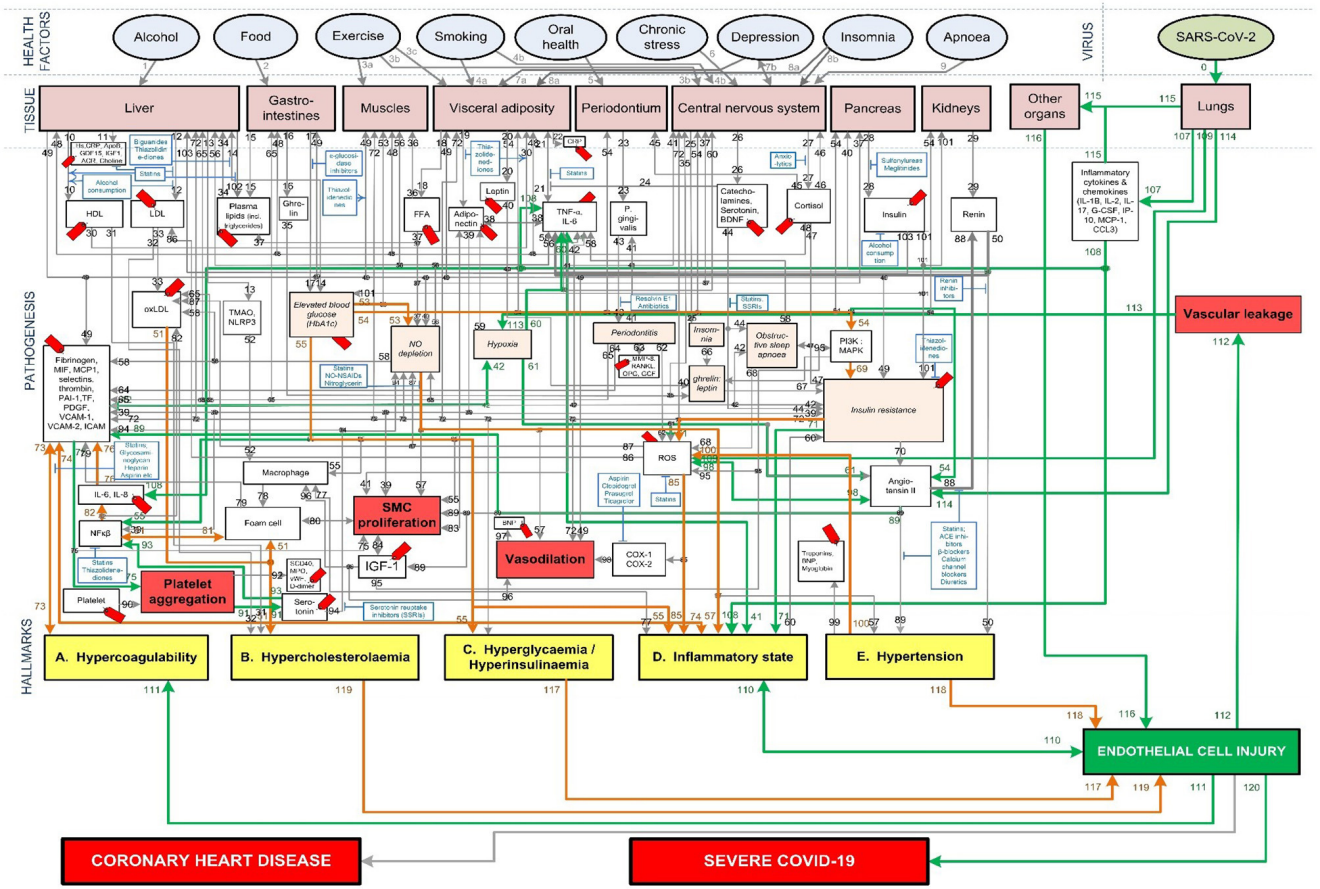
Proposed integrated Covid-19/CHD model.

**Figure 5:**
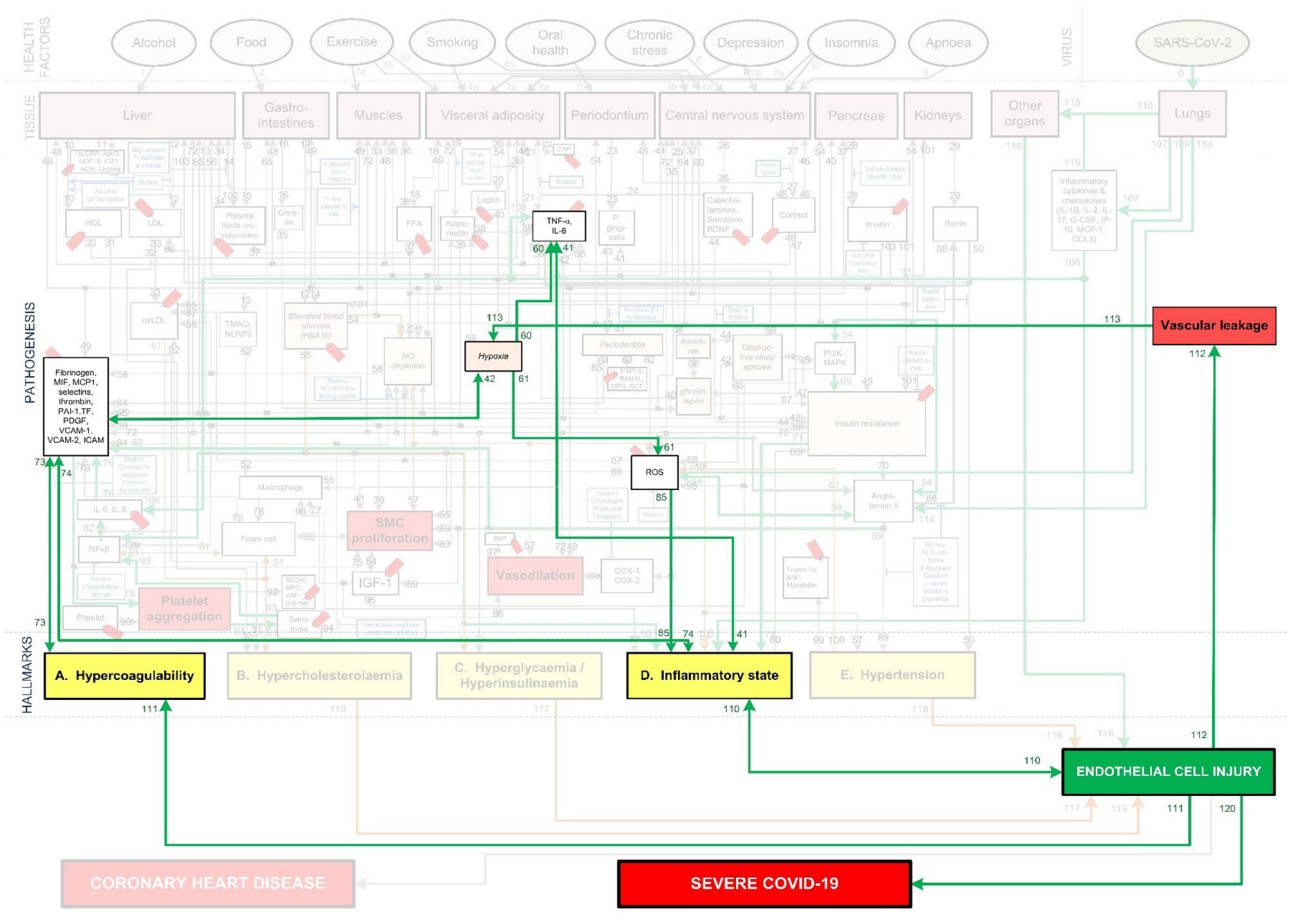
*Death spiral* evident in some critical Covid-19 patients.

**Figure 6:**
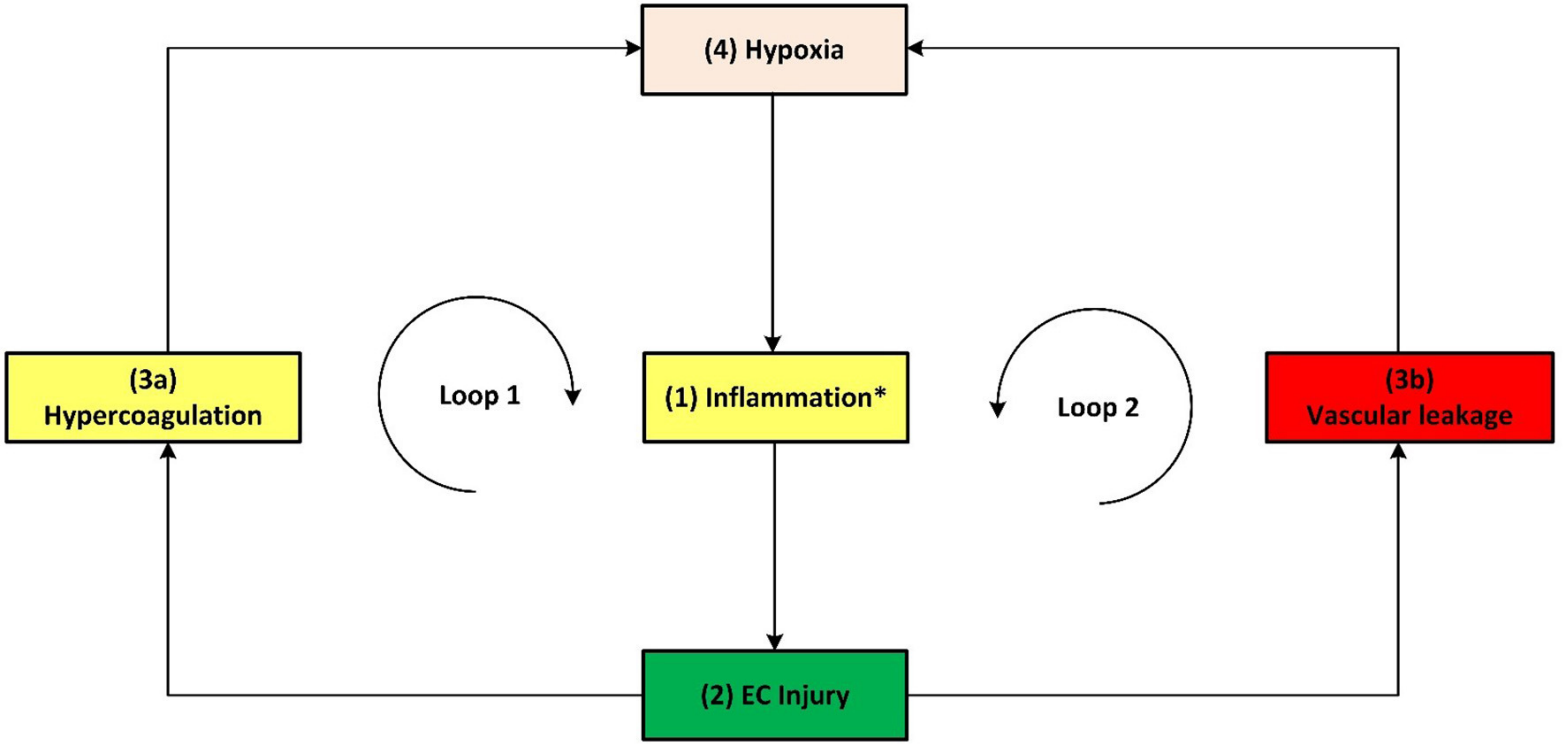
Simplified schematic of the *death spiral* evident in some critical Covid-19 patients. The *death spiral* can be summarized as follows: Increased (1) inflammation at the lungs causes (2) EC injury, which can result in activation of the (3a) coagulation cascade and/or (3b) vascular leakage at the lungs, thereby causing (4) hypoxia which further increases inflammation, creating two closed positive feedback loops and causing severe Covid-19 through a *death spiral*. *As described in the text, this inflammation is initiated by various factors, primarily by a hyperimmune response to infection of SARS-CoV-2 (cytokine storm) but also other factors such as hyperinsulinaemia/hyperglycaemia or hypercholesterolaemia.

The methodology to develop the pathogenic pathways for the *integrated CHD/Covid-19 model* is divided into three parts namely the following:

1. Section Description of Existing CHD Model discribes the existing CHD model (23).
2. Section Systems-Based Integration of Covid-19 Factors Into the CHD Model discusses the systems-based method for integration (Figure 2) of Covid-19 factors into the CHD model (Figure 1). The outcome of this method is depicted in Figures 3–6, **8**–**10**. Its implications are discussed in the Results sections Integrated Covid-19/CHD Model and Covid-19 Aggravation in Patients With Pre-existing CHD Comorbidities.
3. Section Evaluation of *Health Factors* and *Pharmaceutical Interventions* describes the method to evaluate the effects on Covid-19 severity of *health factors* (blue ovals) and *pharmaceutical interventions* (blue boxes) as depicted in Figure 1. The relevant pathogenic pathways that are activated are discussed in more detail in the Results sections Effects of Different *Health Factors* on Covid-19 Severity and Effects of Different CHD *Pharmaceutical Interventions* on Covid-19 Severity.

### Description of Existing CHD Model

The existing CHD model (Figure 1) was developed as a PhD study and extensively described in (23). The model is available online from the university (23). Some results and implications of the model were published (24–28). Hence, we will only discuss the relevant salient elements here. The model defined CHD as the incidence of atherosclerosis, coronary artery disease, or myocardial infarction (23). Subsequently, where results were given for cardiovascular disease these were interpreted as CHD only in scenarios where the effect of stroke could be accounted for (23).

Although cerebrovascular disease is also a component of cardiovascular disease it was not addressed here. Our proposed *integrated CHD/Covid-19 model* is therefore based primarily on CHD attributes, with focus on vascular complications induced by the SARS-CoV-2 virus. We acknowledge that other pathogenic pathways may exist such as the cerebrovascular ones (35), which should warrant further research in an extended model.

The CHD model presented in Figure 1, was developed by analyzing the effect of different *health factors* (blue ovals) on *body tissues* (pink boxes) and investigating the respective *pathogenesis* (gray lines with numbers), *traits* (orange boxes) and activated *biomarkers* (white boxes) related to an increased risk of CHD (23–28).

Each gray line and respective number in the CHD model correspond to a certain pathogenesis pathway that could typically be present in a CHD patient. These pathways are visual representations of previously published literature, which link the effects of *health factors* (blue ovals) to the relevant *tissues* (pink boxes) and subsequently to the hallmarks of CHD (yellow boxes) (23–28).

The traits are represented in the lightly shaded orange boxes. Biomarkers are indicated as white boxes, with those that are typically measured, denoted with red tags *(*
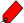
*)*. The *pharmaceutical interventions*, acting on the respective pathways are indicated as blue boxes, where blunted blue arrows *(*
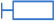
*)* denote antagonize or inhibit and pointed blue arrows *(*
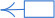
*)* denote up-regulate or facilitate (23–28).

### Systems-Based Integration of Covid-19 Factors Into the CHD Model

The systems-based integration methodology dicussed here is depicted as three phases in a flow chart in Figure 2. The iterative approach followed here is to ensure that only pathways discussed in literature, with substantial evidence, are included. We will use Figure 3 to show the Covid-19 pathways in green, with all other pathways from the original model in Figure 1 made transparent.

#### Phase 1: SARS-CoV-2 and CHD

SARS-CoV-2, which causes Covid-19, was incorporated into the existing CHD model by investigating pathogenic pathways and biomarkers reported in literature. These biomarkers and pathways were either included or excluded based on the following five steps, presented in Figure 2 (Phase 1).

**Step (1):** Firstly, the relevant tissue (denoted as pink boxes in the right-hand corner of Figure 3) through which the SARS-CoV-2 virus (green oval in Figure 3) enters the body was evaluated. Although EC injury was discussed as the critical element in CHD in (23), it was not shown in Figure 1. Here we added EC injury as a green box between pathways 110, 111, 112, and 116 at the bottom of Figure 3.

**Step (2):** The activated CHD related biomarkers, traits or hallmarks, reported in severe Covid-19 patients were then identified from literature. These are, respectively, denoted as white, orange and yellow boxes in Figure 3.

**Step (3):** In this step we evaluated the identified CHD biomarkers, traits or hallmarks found in literature, in order to determine whether the activation of these occurs directly or indirectly as a result of the SARS-CoV-2 virus. Steps (4) and (5) describe the two possible outcomes of the identification process.

**Step (4):** If the activation occurs directly, as determined in step (3), then a new (green) pathway that led from the virus to the respective CHD biomarker, trait or hallmark was added to the integrated model as shown in Figure 3.

**Step (5):** If the activation occurs indirectly, as determined in step (3), then a new biomarker or trait was added to the model e.g., the inflammatory cytokines in the top, right-hand white box between pathways 107, 108, and 115 in Figure 3. A biomarker or trait was only added if its respective pathway eventually led to the activation of a CHD hallmark.

#### Phase 2: Iteration

For the iteration process in Phase 2, the following steps were conducted:

**Step (6):** The activated component (CHD hallmark, biomarker or trait) from step (4) to which the green pathway from step (4) leads was further evaluated based on literature. If this component is a biomarker or trait then step (7) was followed. If this component is rather a CHD hallmark, then step (12) was followed.

**Step (7):** In this step it was determined whether the CHD biomarker or trait has any outgoing (gray) CHD pathways. Most biomarkers and traits have outgoing CHD pathways. These gray CHD pathways were further assessed in Step (8). For the biomarkers and traits with no outgoing gray pathways (e.g., troponin for pathway 99 in Figure 3) step (11) was followed.

**Step (8):** In this step it was determined whether the gray CHD pathway leads directly or indirectly to a CHD hallmark (yellow boxes in Figure 3). If the gray CHD pathway leads directly to a CHD hallmark then step (9) was followed, otherwise step (10) was followed.

**Step (9):** The CHD hallmark was further investigated to ensure its activation due to SARS-CoV-2 was relevant to severe Covid-19 patients. If it was reported in literature to be aggravated in severe Covid-19 patients then step (12) was followed (changing the gray pathway to a green pathway) otherwise step (11) was followed (keeping the pathway gray). These steps are explained in more detail in phase 3.

**Step (10):** As determined in step (8), the relevance to Covid-19 severity of the subsequent CHD biomarker or trait to which the gray CHD pathway led to was investigated. If relevance was found, then this CHD biomarker or trait was re-evaluated by following the same approach as in step (7).

#### Phase 3: Outcome

This phase presents the two outcomes that were reached after integration and iteration of the identified biomarkers, traits, CHD hallmarks and their relevant pathways.

**Step (11):** This step was followed if the activated CHD biomarkers or traits had, (i) no other outgoing CHD pathway or (ii) the outgoing pathway led to another biomarker or trait that had no relevance to severe Covid-19 patients. If one of these two conditions were met then the biomarker, trait and the subsequent pathway was not evaluated further.

These biomarkers, traits and respective pathways e.g., oxidized low-density lipoprotein (oxLDL), nitric oxide (NO) depletion and cortisol were made transparent, as shown in Figure 3. Although these biomarkers or traits do not have a direct link to Covid-19 patients, they may influence Covid-19 severity indirectly by affecting one of the CHD hallmarks. This idea is discussed in more detail in section Covid-19 Aggravation in Patients With Pre-existing CHD Comorbidities.

**Step (12):** Step (12) was followed if the investigated biomarker, trait, CHD hallmark and respective pathways were relevant in most Covid-19 patients with severe disease and these are therefore prominently shown as green lines in Figure 3.

The Covid-19 pathways were described in this section and shown as green lines in Figure 3. The final step is to show all the CHD pathways together with the Covid-19 pathways. The complete *integrated CHD/Covid-19 model* is given in Figure 4.

### Evaluation of *Health Factors* and *Pharmaceutical Interventions*

The mechanisms of interaction between CHD and Covid-19 (Figure 4) can help to compare the few factors a patient can control namely, *health factors* (before infection with SARS-CoV-2) and *pharmaceutical interventions* (after infection). Only the *health factors* and *pharmaceutical interventions* investigated in (23) for CHD risk are investigated here for Covid-19.

The *health factors* (blue ovals) in Figure 4 were defined as the following (23):

- Alcohol use: Indicates moderate alcohol consumption (20–30 g alcohol (ethanol) per day for men and half of that for women).
- Food: High glycemic diets (HGD) (glycemic load > 142).
- Exercise: Regular moderate exercise (550–3,000 kcal/week).
- Smoking: Current smoker.
- Oral Health: oor oral health in the form of periodontal disease.
- Stress: Chronic-level stress at work or home.
- Depression: Self-diagnosed, physician diagnosed or use of antidepressant medication.
- Insomnia: Inability to fall asleep or to maintain sleep or the perception of disturbed sleep.
- Apnoea: Obstructive sleep apnoea or hypopnoea (apnoea-hypopnea index>5/h).

We will discuss in Results section Effects of Different *Health Factors* on Covid-19 Severity to what extent a healthy vascular “baseline,” as a result of a healthy lifestyle, will influence Covid-19 severity.

The *pharmaceutical interventions* that were investigated were limited to those investigated in the original CHD model (23). These include statins, salicylates (aspirin), indirect thrombin inhibitors (heparin), direct thrombin inhibitors (angiomax), Angiotensin-Converting-Enzyme (ACE) inhibitors, angiotensin-renin inhibitors, β-blockers, calcium channel blockers, diuretics, biguanides (metformin) and antidepressants. They are indicated in Figure 1 as blue boxes, where blunted blue arrows *(*
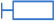
*)* denote antagonize or inhibit and pointed blue arrows *(*
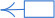
*)* denote up-regulate or facilitate.

Although larger studies of how the *health factors* and *pharmaceutical interventions* influence a person's risk for CHD are usually available, Covid-19 data are often limited. Nevertheless, several studies exist that evaluated the effect of many *health factors* and *pharmaceutical interventions* on Covid-19 severity. Limitations of these studies are that they vary in study size and design i.e., some studies are case-control studies hence only reporting odds ratio (OR), whereas others are cohort studies or clinical trials that report on relative risks (RR) or hazard ratios (HR).

Unfortunately, RR, HR and OR are not the same and should only be compared in cases where the event being assessed is rare in the control group. In other words, the baseline risk of the control group should approximately be zero. However, at present it is the best information we have. Until better data becomes available, these studies were used as an initial indicative comparison between the effect what *health factors* and *pharmaceutical interventions* have on CHD risk and Covid-19 severity. This also applies to the data used to compare the risk between coagulation and Covid-19 severity in section The Death Spiral: Inflammation, EC Injury, Coagulation, Vascular Leakage and Hypoxia.

In this paper the comparison of the data between CHD risk and Covid-19 severity was graphically reported using a non-traditional method (23–28). The risks that indicate an increase in disease severity are displayed as reported, whereas the risk values that show a decrease in severity are presented as the inverse of the reported value.

This method presents a better visual illustration when comparing an increase and decreased risk. For example, a conventional RR = 3 constitutes to a 3-fold increase in risk while a RR = 0.33 constitutes to a 3-fold decrease in risk (1/0.33 = 3). The method has also been used in previous papers (24–28).

## Results

Section Integrated Covid-19/CHD Model discusses Figures 3, 5, 6 in detail illustrating the detrimental interplay between inflammation, EC injury, coagulation and hypoxia. This visually explains the *death spiral* seen in some Covid-19 patients.

Section Covid-19 Aggravation in Patients With Pre-existing CHD Comorbidities discusses how each pre-existing CHD comorbidity/hallmark could further aggravate this *death spiral. Five* figures are provided (Figures 5, 6, **8**–**10**). These figures illustrate how patients with pre-existing *Hypercholesterolemia* (**Figure 8**), *Hyperglycemia/Hyperinsulinemia* (**Figure 9**) or *Hypertension* (**Figure 10**) could aggravate this *death spiral*. Note that Figures 3, 5, 6, **8**–**10** are simplified versions of Figure 4 (the fully *integrated CHD/Covid-19 model*). Only the prominent pathways, which are needed to explain a specific phenomenon, are shown in these Figures.

In sections Effects of Different *Health Factors* on Covid-19 Severity and Effects of Different CHD *Pharmaceutical Interventions* on Covid-19 Severity the effects that *health factors* and *pharmaceutical interventions* have on developing severe Covid-19 are discussed with reference to the model in Figure 4.

### Integrated Covid-19/CHD Model

#### EC Injury From SARS-CoV-2 Viral Infection

Cell entry and pathologic effects of the SARS-CoV-2 virus mostly occur through two pathways namely, (i) the mucous membranes (primarily infecting the nasal epithelia) or (ii) the respiratory tract (infecting respiratory epithelial cells) (36). This infection typically occurs *via* ACE2 (36), which partially decreases ACE2 function. This leads to an upregulation of angiotensin II effects, including among others an enhanced *Inflammatory* response (17, 36), increased EC injury (37) and state of *Hypercoagulability* seen in severe Covid-19 patients (4, 6–9).

These effects are illustrated in Figure 3 by following the relevant pathways (green lines with numbers) from SARS-CoV-2 (green oval) to the respective biomarkers (white boxes) or traits (orange boxes) and/or hallmarks (yellow boxes). The model will be interpreted in the following way for the rest of this paper:

a) Evidence from literature describing the pathogenesis with the respective (references).
b) These relevant pathways in Figures 3–6, **8**, **9** are then given to illustrate the pathogenesis. Each pathway starts with the relevant tissue, biomarker or trait.
c) The relevant pathway (pw) numbers (#) are denoted as (pw#) e.g., pathway 112 (pw112) links EC injury with vascular leakage.
d) The upwards arrow (↑) represents an upregulation of the respective biomarker/trait/hallmark while the downwards arrow (↓) represents a downregulation.

Figure 3 illustrates how viral infection from SARS-CoV-2 may lead to an activation of a pro-inflammatory state, which causes EC injury *via* the following process:

- Angiotensin II can downregulate phosphoinositide 3-kinase (PI3K) pathway, which increases insulin resistance that directly effects inflammatory state (38). The relevant pathways in Figure 3 are: SARS-CoV-2 viral infection within the lungs *via* (pw0), which through (pw114) upregulates angiotensin II. This follows a downregulation of biomarker PI3K *via* (pw54) that increases insulin resistance through (pw69). This leads to a pro-inflammatory state *via* (pw71), which, through (pw110), results in EC injury. The notation for this pathway and the rest of the paper will be as follows: *SARS-CoV-2-(pw0)-Lungs-(pw114)-*↑*angiotensin II-(pw54)-*↓*PI3K-(pw69)-*↑*insulin resistance-(pw71)-*↑*inflammatory state-(pw110)-*↑*EC injury*.
- Angiotensin II can also upregulate various reactive oxygen species (ROS) at the site of infection, which causes a heightened inflammatory response (38). See Figure 3 pathways: *SARS-CoV-2-(pw0)-Lungs-(pw114)-*↑*angiotensin II-(pw98)-*↑*ROS-(pw85)-*↑*inflammatory state-(pw110)-*↑*EC injury*.
- An upregulation of angiotensin II may increase platelet factors, which increases the risk for coagulability (38, 39). Since hypercoagulation and inflammation are interrelated, an inflammatory state may be enhanced (39). See Figure 3 pathways: *SARS-CoV-2-(pw0)-Lungs-(pw114)-*↑*angiotensin II-(pw89)-*↑*platelet factors-(pw73)-*↑*Hypercoagulability-(pw73)-(pw74)-*↑*inflammatory state-(pw110)-*↑*EC injury*.
- Furthermore, an increase in platelet factors can also upregulate platelet aggregation (38). This could increase the inflammatory mediator nuclear factor-kappa-beta (NFκβ), aggregating inflammation (38). See Figure 3 pathways: *SARS-CoV-2-(pw0)-Lungs-(pw114)-*↑*angiotensin II-(pw89)-*↑*platelet factors-(pw75)-*↑*platelet aggregation-(pw91)-serotonin-(pw93)-*↑*NFk*β*-(pw55)-*↑*inflammatory state-(pw110)-*↑*EC injury*.

In addition to this pro-inflammatory state that causes EC injury, the virus can also directly cause EC injury in other organs. This could happen if the virus enters the bloodstream and binds to ACE2 receptors located in other organs (9). Considerable evidence shows that the lungs of patients who died from Covid-19, have severe EC injury (endothelialitis) associated with the presence of intracellular viral infection (4). The presence of viral particles were also found in the ECs of the liver, kidneys and heart (9, 40). This could then lead to inflammation and EC damage at the infected organ. See Figure 3 pathways: *SARS-CoV-2-(pw0)-Lungs-(pw115)-infect other organs via blood-(pw116)-*↑*EC injury*.

#### EC Injury From a Hyperimmune Response to Infection

Infection from SARS-CoV-2 causes damage-associated molecular patterns to occur, which can trigger a hyperimmune response. Most severe cases of patients with Covid-19 display a defective hyperinflammatory state with significantly increased serum levels of pro-inflammatory cytokines and chemokines (41–44).

This overproduction of pro-inflammatory cytokines and chemokines can damage lung infrastructure and further induce EC injury of pulmonary blood vessels (17, 20, 45), see Figure 3 pathways: *SARS-CoV-2-(pw0)-Lungs-(pw107)-*↑*pro-inflammatory cytokines & chemokines-(pw108)-*↑*inflammatory state-(pw110)-*↑*EC injury*.

Most critical cases show increased levels of, among others, the pro-inflammatory cytokines interleukin-6 (IL-6), interleukin-8 (IL-8) and tumor necrosis factor-α (TNF-α) (41–43). These pro-inflammatory cytokines directly cause an upregulation of inflammation (45). See Figure 3 pathways: *SARS-CoV-2-(pw0)-Lungs-(pw107)-*↑*pro-inflammatory cytokines-(pw108)-*↑*TNF-*α*, IL-6-(pw41)-*↑*inflammatory state-(pw110)-*↑*EC injury*.

These cytokines can also indirectly upregulate inflammation through dysregulation of platelet factors (46). See Figure 3 pathways: *SARS-CoV-2-(pw0)-Lungs-(pw107)-*↑*pro-inflammatory cytokines-(pw108)-*↑*IL-6, IL-8-(pw76)-*↑*platelet factors-(pw74)-*↑*inflammatory state-(pw110)-*↑*EC injury*.

Furthermore, a hyperinflammatory state induced by an unmodulated immune response can also cause EC injury. This happens when neutrophils activate pathways that elevate reactive oxygen species (ROS) (22, 47). See Figure 3 pathways: *SARS-CoV-2-(pw0)-Lungs-(pw109)-*↑*ROS-(pw85)-*↑*inflammatory state-(pw110)-*↑*EC injury*.

A hyperinflammatory response of cytokines can circulate to other organs. This could lead to acute inflammation such as septic shock and/or multiple organ damage, which may further cause EC injury (48). See Figure 3 pathways: *SARS-CoV-2-(pw0)-Lungs-(pw107)-pro-inflammatory cytokines & chemokines-(pw115)-other organs-(pw116)-*↑*EC injury*.

#### The Death Spiral: Inflammation, EC Injury, Coagulation, Vascular Leakage and Hypoxia

Note that *hypoxia* shown in Figures 1, 3–6 includes hypoxemia. Although hypoxia might be respiratory related, vascular related EC injury could be one of the main factors fueling this hypoxia (45, 49, 50). This vascular related hypoxia may result from either hypercoagulation or vascular leakage, both stemming from EC injury (17, 22, 45, 46, 49, 50).

Vascular leakage from EC injury leads to an increase in leucocytes and platelets as well as vascular permeability (50). This results in fluid from the blood to enter the alveoli, filling the alveolar space. In turn it decreases the efficiency of gas exchange in the lungs (50). This prevents the body from taking in sufficient oxygen, leading to different severity levels of hypoxia (50). These pathways are denoted in Figure 5 as: *EC injury-(pw112)-*↑vascular leakage*-(pw113)-*↑*hypoxia*.

On the other hand, coagulation stemming from EC injury articulates glycoproteins that are involved in hemostasis, to which platelets bind. This consequently upregulates the expression of platelet tissue factors, which are the prime activators of a coagulation cascade (22, 51). This leads to a high possibility of disseminated intravascular coagulation, congestion of the small capillaries by inflammatory cells and thrombosis in larger vessels (45).

Congestion or clogging of pulmonary blood vessels could increase hypoxemia *via* ventilation/perfusion mismatch and low level of mixed venous blood oxygen (49). This build-up of blood clots in blood vessels within the lungs are commonly found in critically ill and non-surviving Covid-19 patients (6, 21, 52). These pathways are denoted in Figure 5 as: *EC injury-(pw111)-*↑Hypercoagulability*-(pw73)-*↑*platelet factors-(pw42)-*↑*hypoxia*. Hypoxia also results in further upregulation of inflammation by activating IL-6 & TNF-α (53) or increasing ROS leading to further EC injury (54). See Figure 5 pathways: *Hypoxia-(pw60)-*↑*TNF-*α*, IL*-6*-(pw41)-*↑*inflammatory state* or *Hypoxia-(pw61)-*↑*ROS-(pw85)-*↑*inflammatory state-(pw110)- EC injury*.

With the aforementioned knowledge a summary of the main pathogeneses describing the *death spiral* are given. Note that inflammation has two different outgoing pathways (loops) that can lead to increased hypoxia. Both pathways are denoted in Figure 5 as follows:

1. **Hypercoagulability (positive feedback loop 1):** Inflammation from Covid-19 results in EC injury which may activate the coagulation cascade, forming microthrombi in the blood vessels near the alveoli (4, 6–9). This reduces oxygenation efficiency, see pathways: ↑*inflammatory state –(pw110)-EC injury-(pw111)-*↑*Hypercoagulability-(pw73)-*↑*platelet factors-(pw42)-*↑*hypoxia-(pw60)-*↑*TNF-*α*, IL-6-(pw41) AND/OR (pw61)-*↑*ROS-(pw85)-*↑*inflammatory state-(pw110)-Loop repeated-(pw120)-Severe Covid-19*.
2. **Vascular leakage (positive feedback loop 2):** Inflammation from Covid-19 results in EC injury. EC injury in blood vessels near the alveoli can lead to vascular leakage (22). This causes fluid build-up within the alveoli (50), subsequently reducing oxygenation efficiency, see pathways: ↑*inflammatory state –(pw110)-EC injury-(pw112)-*↑*vascular leakage-(pw113)-*↑*hypoxia-(pw60)-*↑*TNF-*α*, IL-6-(pw41) AND/OR (pw61)-*↑*ROS-(pw85)-*↑*inflammatory state-(pw110)-Loop repeated-(pw120)-Severe Covid-19*.

A simplified schematic of the *death spiral* is illustrated in Figure 6, which shows the two closed positive feedback loops leading to hypoxia. If a Covid-19 patient becomes hypoxic, it is important to break these loops by administering supplemental oxygen. This is currently done in practice where supplemental oxygen reduces disease severity in hypoxic Covid-19 patients (55).

To reduce the risk of developing hypoxia one should focus on reducing inflammation that leads to the downstream effects namely EC injury, coagulation and vascular leakage. This is also seen in practice where various pharmaceutical interventions that treat inflammation have shown promising results e.g., corticosteroid dexamethasone in later stage of illness (56) and anti-inflammatory drugs [Celebrex (57) and aspirin (58)].

If we focus on loop 1 it is expected that people who have a higher risk of developing blood clots (coagulation) should have a higher risk of developing severe Covid-19. There are several uncontrollable factors that are known to increase a person's risk of developing blood clots namely, gender, age, ethnicity, blood type and pregnancy.

**Figure 7:**
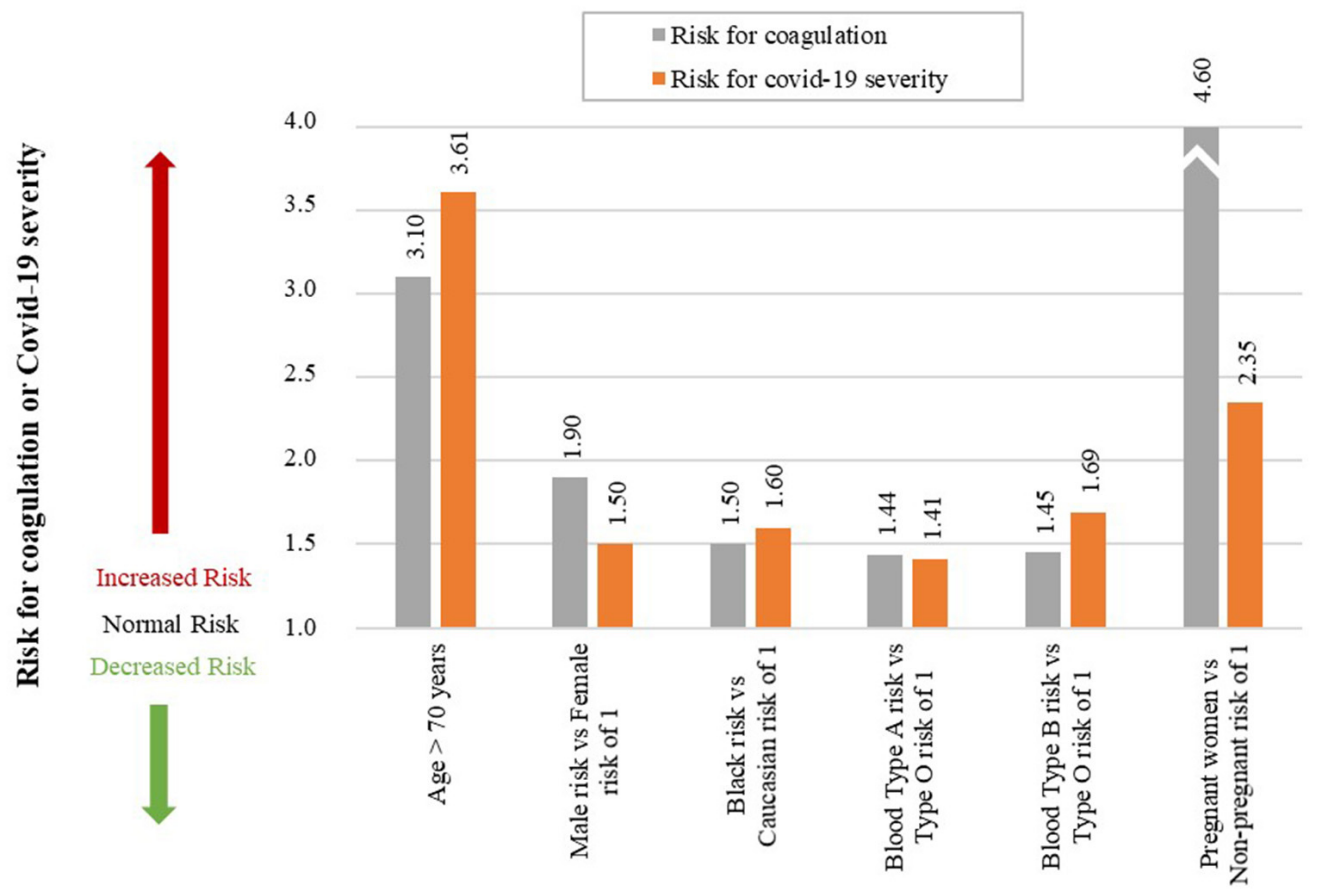
Qualitative comparison of risk factors between coagulation and Covid-19 severity. (An accurate quantitative comparison is not possible, mostly due to differences in study design and size).

Although this does not help the patient, it is of interest to help understand Covid-19 severity in these individuals. The data for the risk of coagulation (blood clots) and Covid-19 severity for these individuals are given in Table 1. A qualitative graphical comparison between the data for coagulation and Covid-19 severity from Table 1 is given in Figure 7.

**Table 1:** Data for the qualitative comparison of risk factors between coagulation and Covid-19 severity.

**Uncontrollable factor**	**Risk for coagulation**					**Risk of covid-19 severity**				
	**Study size (*n* = no. of participants, *N* = no. of studies)**	**RR/OR**	**Value**	**95% CI**	**References**	**Study size (*n* = no. of participants, *N* = no. of studies)**	**RR/OR**	**Value**	**95% CI**	**References**
Age > 70 years	*n* = 607, *N* = 1	OR	3.10	1.3–7.5	(59)	*n* = 36 470, *N* = 59	RR	3.61	2.70–4.84	(60)
Male vs. Female	*n* = 11 253, *N* = 1	RR	1.90	1.9–2.4	(61)	*n* = 36 470, *N* = 59	RR	1.50	1.18–1.91	(60)
Black vs. Caucasian	^#^	RR	1.50	^#^	(62)	*n* = 505 992, *N* = 1	OR	1.60	1.2–2.0	(63)
Blood Type A vs. Type O	*n* = 406 755, *N* = 1	HR	1.44	1.39–1.50	(64)	*n* = 31 100, *N* = 4	OR	1.41	^*^	(65)
Blood Type B vs. Type O	*n* = 406 755, *N* = 1	HR	1.45	1.37–1.54	(64)	*n* = 31 100, *N* = 4	OR	1.69	^*^	(65)
Pregnant vs. Non-pregnant	*n* = 1 142, *N* = 1	OR	4.60	2.7–7.8	(66)	*n* = 22 493, *N* = 1	OR	2.35	1.48–3.74	(67)

*1. CI, Confidence Interval; HR, Hazard Ratio; OR, Odds Ratio; RR, Relative Risk*.

*2*.

(#) *Denotes that the study did not provide this data*.

*3*.

(*) *Study (65) only provides the 95% CI for each Blood Type separately and not the Blood Type vs. Blood Type O. These individual 95% CI's for Blood Type A, B, and O were (1.11–1.40), (0.99–1.21), and (0.63–0.77), respectively. These data were not included in the table since the OR's for each Blood Type were reported separately. Here we normalized the OR's of Blood Type A vs. O and Blood Type B vs. O*.

##### Age

Age is an independent risk factor of coagulation, with thrombotic incidences increasing rapidly in people older than 70 years (59). The odds of venous thromboembolism in a person older than 70 years is three times higher than a person young than 70 years, OR of 3.1 (59).

If we investigate Covid-19 mortality data, a similar trend is seen with age. Risk of mortality due to Covid-19 is much higher in older patients with a RR of 3.61 in patients older than 70 years (60), see Figure 7. The increased risk of coagulation due to older age could be one reason for this increased Covid-19 mortality.

##### Gender

A 25-year population-based study showed that males have a higher risk to coagulate than females (68). At younger ages (<45 years) females have a higher risk of coagulation than males, for various reproductive reasons (61). However, since an increase in Covid-19 severity and mortality is typically seen in older patients (> 45 years) we only focused on these older patients. Men have a 1.9-fold higher risk of developing venous thrombosis than women (61).

Covid-19 data also indicate that males have a higher risk of Covid-19 mortality than females, with a RR of 1.50 (60), see Figure 7. The increased risk of coagulation due to gender for individuals older than 45 years could be one reason for this increased Covid-19 mortality.

##### Ethnicity

Ethnicity has also shown to be an independent risk factor for coagulation. The highest risk of thrombosis being in African Americans, with a RR of 1.5 compared to Europeans (62). This is also seen in Covid-19 mortality data, which shows that African American's have a higher odds of death than Europeans, with an OR of 1.6 (63), see Figure 7. The increased risk of coagulation due to ethnicity could be one reason for this increased Covid-19 mortality.

##### Blood Type

Another risk factor that seems to influence the odds of developing a thromboembolic event is a person's blood type. A single cohort study showed that blood types A&B vs. O have higher risk of developing a thromboembolic event, with the following HRs: A vs. O of 1.44, and B vs. O of 1.45 (64), see Figure 7.

A similar trend is seen in the effect of different blood types on Covid-19 severity, with the following ORs: A of 1.06, B of 1.27, O of 0.75 (65). If these values are normalized with respect to blood type O the ORs are the following: A vs. O of 1.41, and B vs. O of 1.69, see Figure 7.

None of the blood group values for Covid-19 severity were statistically significant (65). It is however interesting that this limited study shows that patients with blood type O have lower odds of developing severe Covid-19 than blood types A and B. There is however still controversy regarding correlation between blood type and Covid-19 severity (69).

##### Pregnancy

Pregnancy is not necessarily an uncontrollable factor, but for the duration of being pregnant it is. During pregnancy the risk of venous thrombosis is much higher than for non-pregnant women, with an OR of 4.6 (66), see Figure 7.

Pregnant women are also at a higher risk of developing more severe Covid-19 complications than non-pregnant women, with an OR of 2.35 (67). Fortunately, no significant association between pregnant and non-pregnant women was found for Covid-19 mortality risk (67). This may be due to pregnant women seeking medical attention earlier than non-pregnant women. The higher severity risk could partially be due to the higher risk for coagulation during pregnancy. More research is however needed to validate this.

The above mentioned uncontrollable factors may contribute to the coagulation loop 1 of the *death spiral*. This could help explain why some patients experience accelerated disease severity. However, better studies for Covid-19 in especially different blood groups are needed.

**Figure 8:**
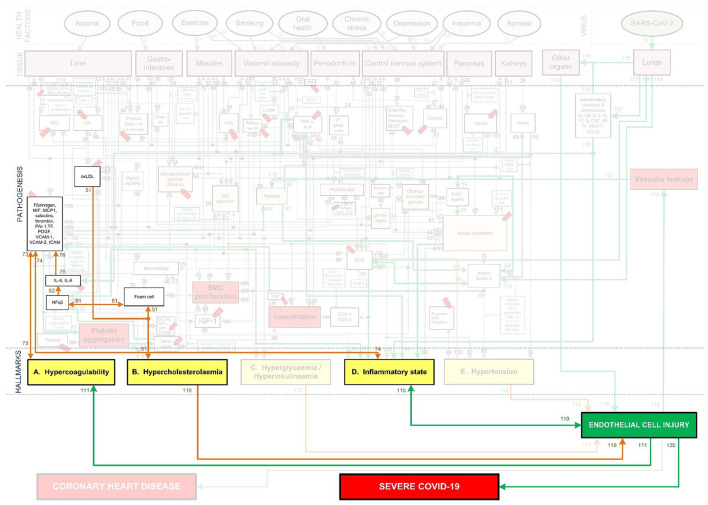
CHD related aggravation of severe Covid-19 in patients with high cholesterol.

**Figure 9:**
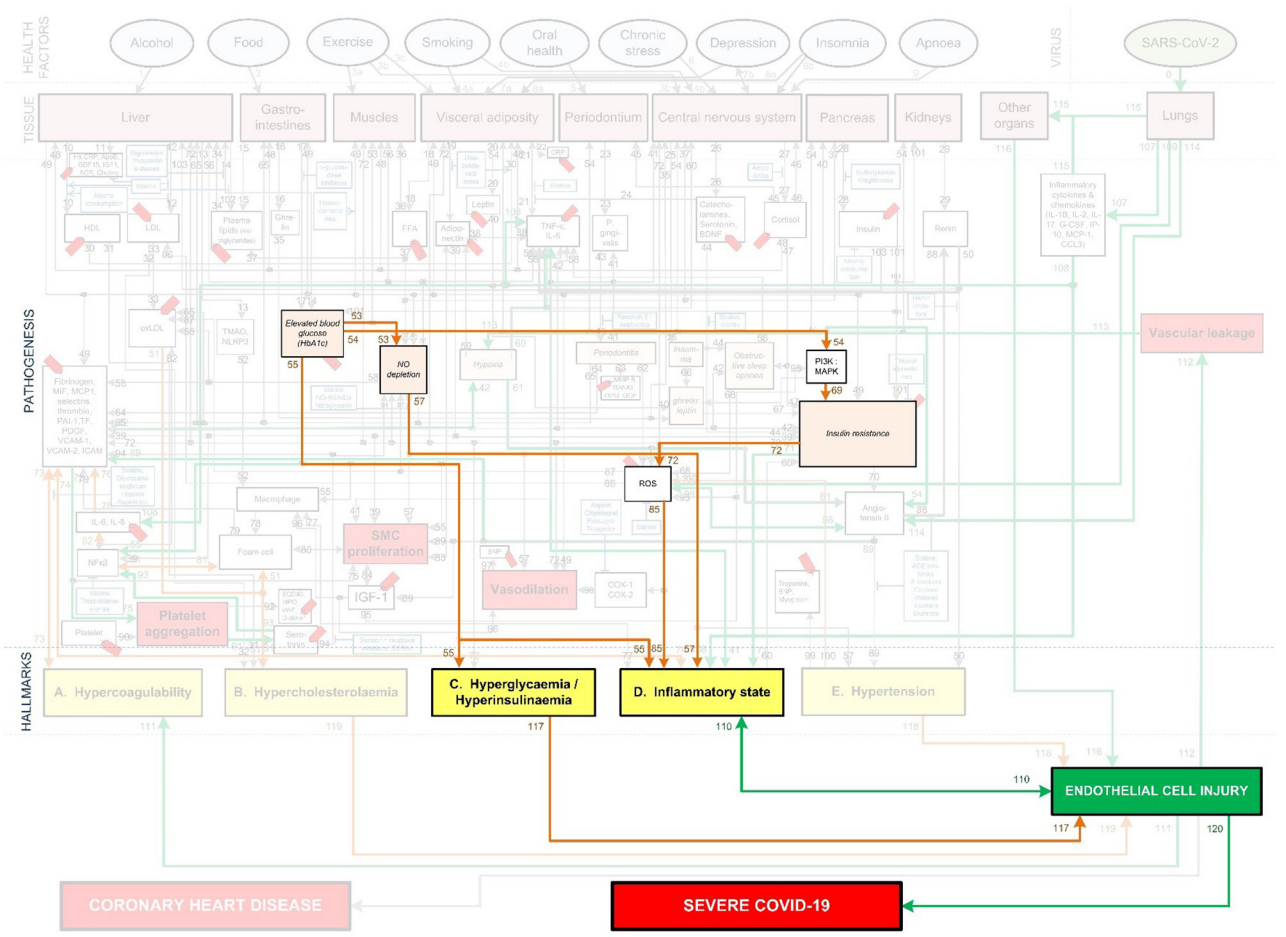
CHD related aggravation of severe Covid-19 in patients with high blood glucose levels.

**Figure 10:**
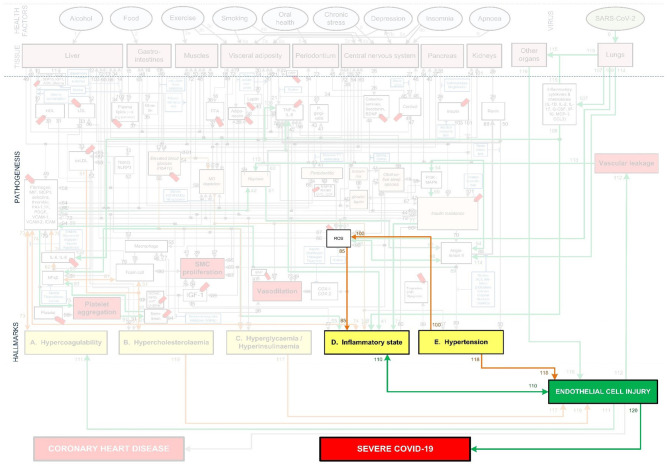
CHD related aggravation of severe Covid-19 in patients with chronic hypertension.

The high mortality statistics in patients with pre-existing CHD comorbidities (10–12, 70) are discussed in more detail in the next Section with reference to Figures 8–10. We will show why a patient with a worse cardiovascular “baseline” before Covid-19 could potentially have a worse outcome than a patient with a healthy cardiovascular “baseline.”

### Covid-19 Aggravation in Patients With Pre-existing CHD Comorbidities

#### Severe Covid-19 Patients With Existing Chronic Hypercholesterolemia

One of the risk factors for CHD is *Hypercholesterolemia*. Chronic *Hypercholesterolemia* may fuel the Covid-19 *death spiral* by increasing the risk of EC injury *via* an inflammatory state or plaque buildup. For EC injury induced by an inflammatory state see Figure 8 pathways: ↑*oxLDL-(pw51)-Hypercholesterolemia-(pw51)-*↑*foam cell-(pw81)-*↑*NFk*β*-(pw82)-*↑*IL-6, IL-8-(pw76)-*↑*platelet factors-(pw74)-*↑*inflammatory state-(pw110)-*↑*EC injury*. For EC injury induced by plaque buildup see Figure 8 pathways: ↑*oxLDL-(pw51)-Hypercholesterolemia-(pw119)-*↑*EC injury*.

*Hypercholesterolemia* could also have an impact on the severity of Covid-19 by increasing coagulation. This could happen by increased foam cell production and increased thrombin generation (29). In turn increasing the platelet forming factors and reducing breakdown processes like fibrinolysis, increases coagulation (30). See Figure 8 pathways: ↑*oxLDL-(pw51)-Hypercholesterolemia-(pw51)-*↑*foam cell-(pw81)-*↑*NFk*β*-(pw82)-*↑*IL-6, IL-8-(pw76)-*↑*platelet factors-(pw73)-*↑*Hypercoagulability*.

The increased coagulation could aggravate thrombi within the lungs and lead to possible hypoxemia (49), potentially cascading the symptoms already experienced by a Covid-19 patient.

The above discussion partially explains why many patients with obesity have a high risk of developing severe Covid-19 complications (71) as obesity is associated with *Hypercholesterolemia* (72, 73).

Interestingly it was also found that free cholesterol, as well as high-and low-density lipoprotein levels are lower in end-stage Covid-19 patients than in patients with less severe Covid-19 (70, 74). Why would cholesterol levels be lower in patients with more severe disease? Could this be explained by the ability of SARS-CoV-2 to use (“consume”) serum cholesterol for its entry into host cells (32).

If this is the case, then high cholesterol levels before infection might enhance viral infection *via* increased availability of serum cholesterol levels but as the virus “consumes” cholesterol the levels would decrease. These facts are however still controversial and further studies are warranted.

#### Severe Covid-19 Patients With Existing Chronic Hyperglycemia or Hyperinsulinemia

Elevated blood glucose aggravates Covid-19 severity and mortality risk irrespective of diabetes (75, 76). One possible reason for this could be the indirect ability of blood glucose to induce EC injury.

Since glucose is the main energy source for cells, any change to its levels could have a direct effect on the cell's metabolism. Changes in blood glucose can cause ECs to undergo apoptosis (cell death or “suicide”), causing the ECs to detach and enter the bloodstream (77). See Figure 9 pathways: *Elevated blood glucose (HbA1c)-(pw55)-Hyperglycemia-(pw117)-*↑*EC injury*. This further leaves behind eroded arteries which activate processes that lead to atherosclerosis, such as smooth cell proliferation (77).

Another pathway through which elevated blood glucose levels contribute to EC injury is through aggravated inflammation. This inflammation is caused by activating the insulin resistance and ROS producing pathways and impaired EC turnover. See Figure 9 pathways: *Elevated blood glucose (HbA1c)-(pw54)-PI3K:MAPK-(pw69)-*↑*Insulin resistance-(pw72)-*↑*ROS-(pw85)-*↑*inflammatory state-(pw110)-*↑*EC injury*. EC turnover is possibly impaired due to accelerated aging or reduced renewal of cells (78, 79). This is most prominent in the microvascular and arterial ECs (80), which may be due to the differences in glucose uptake of cells.

A similar pathway also leads to increased inflammation due to a dysregulation of NO, which plays an important role in controlling the vascular tone and arterial pressure. A decrease in NO prevents ECs from responding to increased glucose stress, which may further accelerate cellular deterioration (79). See Figure 9 pathways: *Elevated blood glucose (HbA1c)-(pw55)-Hyperglycemia-(pw55)-*↑*inflammatory state-(pw110)-EC injury*.

These indirect impacts on EC injury could potentially explain why *Hyperglycemia* is a significant co-morbidity and risk factor for severe Covid-19 patients (70). It highlights the importance of ensuring that the glucose level of a diabetic patient remains within normal ranges. It may also be advantageous to reduce blood glucose levels in non-diabetic patients as elevated glucose in non-diabetic patients also increased Covid-19 severity (75, 76).

#### Severe Covid-19 Patients With Existing Chronic Hypertension

Hypertension is another common co-morbidity in Covid-19 related mortality (81). This could be due to its indirect ability to increase inflammation or the direct injury caused to ECs (82, 83).

The indirect impact occurs through hypertension that increases the amount of ROS, especially from the oxidation of endothelial NO synthesis (83). ROS can impact the inflammatory state and the ECs in several ways. It can, among others, cause EC death and increase the adhesion of inflammatory cells to the normally inert endothelium surface (83). This could potentially exacerbate the response and symptoms related to EC injury. See Figure 10 pathways: *Hypertension-(pw100)-*↑*ROS-(pw85)-*↑*Inflammatory state-(pw110)-*↑*EC injury*.

Chronic hypertension can also directly cause damage to the microvascular ECs (82). High blood pressure strains the ECs and could potentially cause ruptures in plaques that are adhered to the artery wall (82). See Figure 10 pathways: *Hypertension-(pw118)-EC injury*. This creates additional areas that require attention and would probably also increase the inflammatory response.

Existing chronic hypertension can therefore possibly cause injury to the ECs through either the indirect or direct pathways. This injury could potentially contribute to the rapid worsening of health in Covid-19 patients with chronic hypertension (81).

### Effects of Different *Health Factors* on Covid-19 Severity

We discuss the comparison between CHD and severe Covid-19 for different *health factors* with reference to Figure 4. The definition of each *health factor* was given in section Evaluation of *Health Factors* and *Pharmaceutical Interventions*.

Different *health factors* (pink ovals in Figure 4) were originally analyzed in terms of their effects on CHD risk (23). These *health factors* were either associated with an increase or decrease in risk for CHD (23–28). The same *health factors* were investigated for Covid-19 severity. We will show to what extent a healthy CHD “baseline,” as a result of a healthy lifestyle, will influence Covid-19 severity.

**Figure 11:**
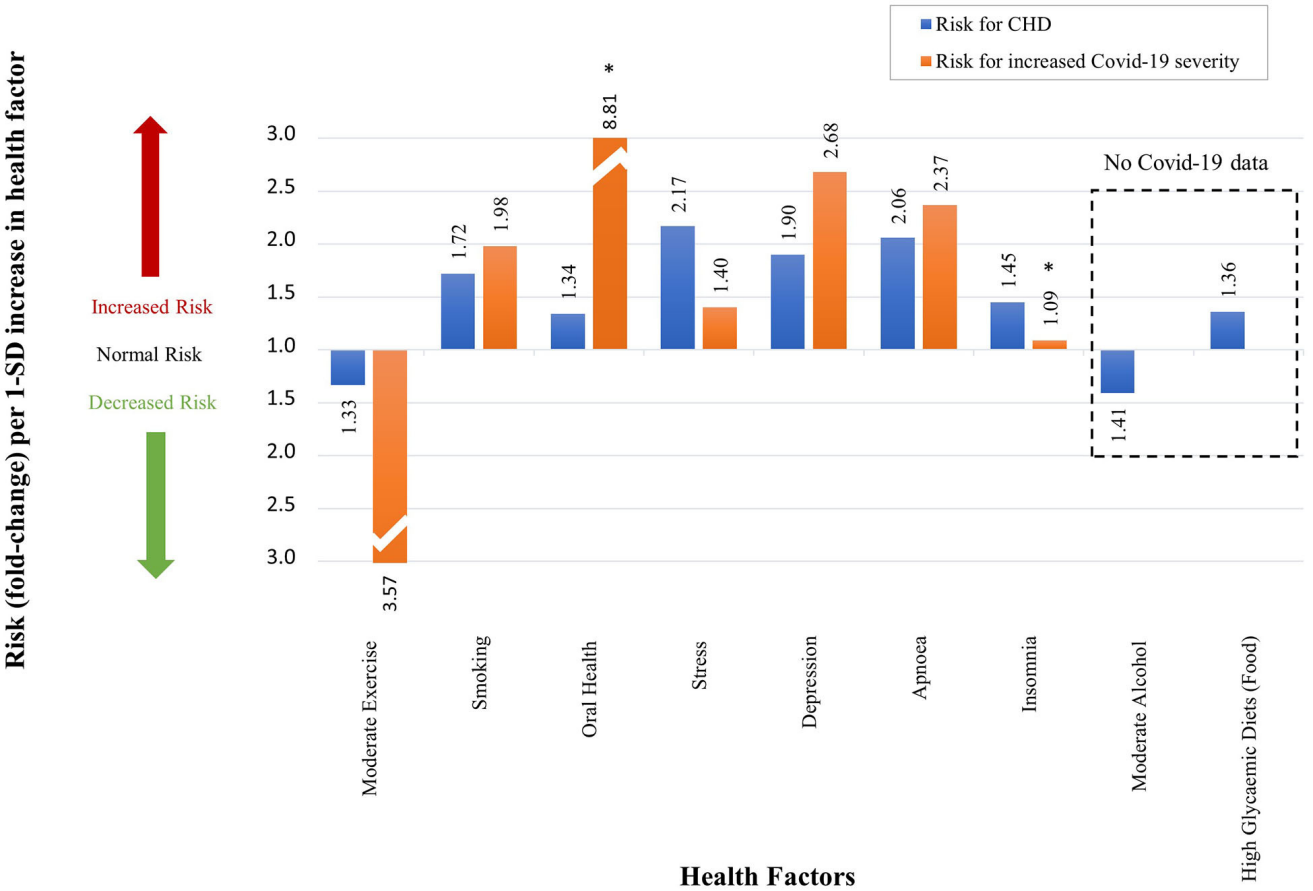
The qualitative effect which different health factors have on CHD risk and Covid-19 severity. (An accurate quantitative comparison is not possible, mostly due to differences in study design and size).

Table 2 summarizes the CHD and Covid-19 data extracted from literature namely, study size (N), number of participants (n), risk type (RR/HR/OR), respective risk value, 95% confidence interval (CI), fold change (as calculated *via* the non-traditional method) and the respective references. Data not statistically significant are indicated with an (^*^) in Figure 11.

**Table 2:** The effect which different health factors and pharmaceuticals have on CHD risk and Covid-19 severity.

	**Risk for CHD**					**References**	**Risk for increased COVID-19 severity**					**References**
	**Study size (*n =* no. of participants, *N =* no. of studies)**	**RR, HR or OR**	**Value**	**95% CI**	**Fold change as per our definition**		**Study size (*n =* no. of participants, *N =* no. of studies)**	**RR, HR or OR**	**Value**	**95% CI**	**Fold change as per our definition**	
**HEALTH FACTORS**												
Moderate exercise	*n =* 645 087, *N =* 33	RR	0.75	(0.71–0.79)	−1.33	(23, 84)	*n =* 260, *N =* 1	OR	0.28	#	−3.57	(85)
Smoking	*n =* 1 010 000, *N =* 141	RR	1.72	(1.62–1.83)	1.74	(23, 86)	*n =* 32 849, *N =* 47	RR	1.98	(1.16–3.38)	1.98	(87)
Oral health	*n =* 147 821, *N =* 7	RR	1.34	(1.27–1.42)	1.34	(23, 27, 88)	*n =* 568, *N =* 1	OR	8.81	(1.00–77.70)	8.81	(89)
Stress	*n =* 24 767, *N =* 1	OR	2.17	(1.84–2.55)	2.17	(23, 90)	*n =* 535, *N =* 1	HR	1.40	(1.11–1.75)	1.40	(91)
Depression	*n =* 124 509, *N =* 21	RR	1.90	(1.49–2.42)	1.90	(23, 92)	*n =* 421 014, *N =* 1	OR	2.68	(2.03–3.54)	2.68	(93)
Apnoea	*n =* 1 436, *N =* 1	HR	2.06	(1.10–3.86)	2.06	(23, 94)	*n =* 15 835, *N =* 4	OR	2.37	(1.14–4.95)	2.37	(95)
Insomnia	*n =* 122 501, *N =* 13	RR	1.45	(1.29–1.62)	1.45	(23, 96)	*n =* 568, *N =* 1	OR	1.09	(0.44–2.71)	1.09	(97)
Moderate alcohol	*n =* 504 651, *N =* 29	RR	0.71	(0.66–0.77)	−1.41	(23, 26, 98)	#	#	#	#	#	#
Food (HGD)	*n =* 220 050, *N =* 8	RR	1.36	(1.13–1.63)	1.36	(23, 25, 99)	#	#	#	#	#	#
**PHARMACEUTICALS**												
Statins	*n =* 169 138, *N =* 26	RR	0.78	(0.76–0.80)	−1.28	(23, 100)	*n =* 13 981, *N =* 1	HR	0.58	(0.43–0.80)	−1.72	(101)
Salicylates (Aspirin)	*n =* 112 000, *N =* 6	RR	0.82	(0.75–0.90)	−1.22	(23, 102)	*n =* 412, *N =* 1	HR	0.53	(0.31–0.90)	−1.89	(58)
Indirect thrombin inhibitors (Heparin)	*n =* 31 402, *N =* 6	OR	0.91	(0.84–0.98)	−1.10	(23, 103)	*n =* 449, *N =* 1	OR	0.37	(0.15–0.90)	−2.70	(104)
Direct thrombin inhibitors (Angiomax)	*n =* 1 883, *N =* 1	HR	0.76	(0.59–0.98)	−1.32	(23, 105)	*n =* 103 703, *N =* 1	HR	0.90	(0.71–1.15)	−1.11	(106)
ACE inhibitors	*n =* 19 141, *N =* 8	OR	0.79	(0.71–0.88)	−1.27	(23, 107)	*n =* 19 486, *N =* 1	HR	0.89	(0.75–1.06)	−1.12	(108)
Angiotensin-renin inhibitors	*n =* 108 212, *N =* 26	OR	0.92	(0.87–0.97)	−1.09	(23, 109)	*n =* 2,877, *N =* 1	RR	0.65	(0.45–0.94)	−1.54	(110)
β-blockers	*n =* 12 825, *N =* 9	RR	0.69	(0.59–0.82)	−1.45	(23, 111)	*n =* 101 141, *N =* 8	OR	1.23	(0.74–2.04)	1.23	(112)
Calcium channel blockers	*n =* 10 136, *N =* 8	OR	0.83	(0.67–1.03)	−1.20	(23, 107)	*n =* 106 566, *N =* 8	OR	0.94	(0.8–1.10)	−1.06	(112)
Diuretics	*n =* 192 478, *N =* 42	RR	0.79	(0.69–0.92)	−1.27	(23, 113)	*n =* 99 669, *N =* 5	OR	0.96	(0.81–1.15)	−1.04	(112)
Biguanides (Metformin)	*n =* 11 385, *N =* 6	OR	0.74	(0.62–0.89)	−1.35	(23, 114)	*n =* 1 800 005, *N =* 1	HR	0.77	(0.73–0.81)	−1.30	(115)
Antidepressants	*n =* 93 653, *N =* 1	HR	0.48	(0.44–0.52)	−2.08	(23, 116)	#	#	#	#	#	#

*CHD, Coronary Heart Disease; CI, Confidence Interval; Covid-19, Coronavirus Disease of 2019; HGD, High Glycemic Diets; HR, Hazard Ratio; OR, Odds Ratio; RR, Relative Risk. A minus sign shows a reduction in risk. (#) denotes that the respective study did not provide this data. A small preliminary study (117) on the effect of the SSRI antidepressant, fluvoxamine, on Covid-19 has shown positive effects. Risk data were not given*.

Where data were unavailable a hash (#) was inserted in Table 2 e.g., for the two *health factors*, alcohol use and food intake (high glycemic diets). These *health factors* have not yet been fully investigated in Covid-19 patients. Despite no risk values being available for these *health factors*, their probable effects on Covid-19 severity are discussed in this section.

The *health factors* that increase/decrease a person's risk for CHD similarly increase/decrease a person's risk (RR/HR/OR) for developing severe Covid-19 (Figure 11). In the rest of this section we will discuss, in more detail, the effects each *health factor* has on the CHD hallmarks, and hypothesize how this could affect Covid-19 severity.

### Moderate Exercise

Based on the CHD model (Figure 1) our research group has published a detailed description of the mechanism by which moderate exercise may reduce CHD risk (28). Only the salient features of the mechanism will be described here.

Regular moderate exercise is universally accepted to reduce the risk of CHD (23, 28, 84) (the definition of moderate exercise was given in section Evaluation of *Health Factors* and *Pharmaceutical Interventions*). Table 2 shows a decrease risk (RR) of 0.75 (*n* = 645 087, *N* = 33) (84). This translates to a 1.33-fold decrease in CHD risk (23, 28) as illustrated in Figure 11.

The effect of moderate exercise on Covid-19 was analyzed in a small cross-sectional study (*n* = 260) (85). The authors concluded that moderate physical activity before onset of Covid-19 decreases the odds of developing severe Covid-19 (OR of 0.28) by 3.57 times (85), see Table 2 and Figure 11. Although this is only a small study, a larger study (*n* = 48 440) substantiates the benefit of regular moderate exercise (118).

This larger study's results are not presented in Table 2 or Figure 11 since the study reported on inactivity. However, since being active helps reduce the odds of developing severe Covid-19, inactivity is expected to have an opposite effect. This is indeed the case as the study showed that patients who are consistently inactivate are 2.49 (OR) times more likely to die from Covid-19 (118).

Therefore, moderate exercise before the onset of disease decreases both the risk for CHD and Covid-19 severity. This could most likely be explained by the effect of moderate exercise on several CHD hallmarks. Moderate exercise largely influences, among others, glucose, cortisol and inflammatory mediator levels (23, 28), therefore reducing the risk of *Hyperglycemia/Hyperinsulinemia* and a heightened *Inflammatory state* (23, 28).

Regular exercise also reduces the accumulation of visceral fat, which reduces the risk of increased Low-Density Lipoprotein (LDL) levels thus decreasing the risk for *Hypercholesterolemia* (23, 28). A decrease of visceral fat also reduces the risk of insulin resistance, which lowers one's risk for increased platelet factors and the potential for *Hypercoagulability* (23, 28).

The potential decrease of these CHD hallmarks could partially explain the benefit of moderate exercise on the reduced risk of Covid-19 severity. The respective CHD hallmark downregulated by exercise and the activated pathways are denoted in Figure 4 as follows:

- **Hyperglycemia/Hyperinsulinemia:** *Moderate exercise-(pw3a)-muscles-(pw53)-*↓*blood glucose-(pw54)-*↓*PI3K:MAPK-(pw69)-*↓*insulin resistance-(pw72)-Hyperglycemia/ Hyperinsulinemia*.
- **Inflammatory state:** *Moderate exercise-(pw3b)-central nervous system-(pw27)-*↓*cortisol-(pw47)-*↓*insulin resistance-(pw70)-*↓*angiotensin II-(pw89)-*↓*hypertension-(pw100)-*↓*ROS-(pw85)-*↓*COX1/2-(pw85)-*↓*Inflammatory state*.
- **Hypercholesterolemia:** *Moderate exercise-(pw3c)-visceral adiposity-(pw18)-*↓*FFA-(pw37)-*↓*plasma lipids-(pw34)-liver-(pw12)-*↓*LDL-(pw33)-*↓*oxLDL-(pw51)-*↓*Hypercholesterolemia*.
- **Hypercoagulability**: *Moderate exercise-(pw3a)-muscles-(pw53)-*↓*blood glucose-(pw54)-*↓*PI3K:MAPK-(pw69)-*↓*insulin resistance-(pw72)-*↓*platelet factors-(pw73)-*↓*Hypercoagulability*.

The potential decrease in four of the five CHD hallmarks due to moderate exercise before onset of Covid-19 (creating a healthier vascular system “baseline”) could partially explain the decreased risk of Covid-19 severity. These beneficial effects of exercise are based on moderate exertion and not heavy exertion. Heavy exertion exercise has the following detrimental effects: transient immune dysfunction, elevated inflammatory biomarkers, and increased risk of upper respiratory tract infections (119). Therefore, exercise exertion is an important factor to consider during the Covid-19 pandemic.

#### Smoking

Smoking is a risk factor for CHD with a RR of 1.72 (86). A recent systematic review and meta-analysis of 47 studies (32 849 hospitalized Covid-19 patients) showed that current smokers have an increased risk of developing severe or critical Covid-19, RR of 1.98 (87).

Most smokers develop insulin resistance and/or Hyperinsulinemia as compared to non-smokers (120, 121). This association may either be due to the lower adiponectin levels or higher cortisol secretion levels seen in current smokers compared to non-smokers (122, 123). This increases a smoker's risk for *Hyperglycemia/Hyperinsulinemia*.

Moreover, most smokers also have higher plasma triglyceride and lower High-Density Lipoprotein (HDL) cholesterol concentrations than non-smokers (121, 124). This increases a smoker's risk of *Hypercholesterolemia*.

Another CHD hallmark that is upregulated in smokers is a heightened *Inflammatory state*. This is due to an upregulation of several inflammatory markers and cytokines such as TNF-α, granulocyte-macrophage colony-stimulating factor (GM-CSF) and monocyte chemoattractant protein (MCP-1) (125).

Smoking also induces an imbalance between various hemostatic molecules in the blood thereby increasing the state of *Hypercoagulability* (126). This may be due to functional changes in clotting factors such as fibrinogen (126).

The associated pathways and respective CHD hallmarks increased by smoking are shown in Figure 4 as the following:

- **Hyperglycemia/Hyperinsulinemia:** *Smoking-(pw4a)-visceral adiposity-(pw19)-*↓*adiponectin-(pw39)-*↑*insulin resistance-(pw72)-* ↑*Hyperglycemia/Hyperinsulinemia. Smoking-(pw4b)-central nervous system-(pw27)-*↑*cortisol-(pw47)-*↑*insulin resistance-(pw72)-* ↑*Hyperglycemia/Hyperinsulinemia*.
- **Hypercholesterolemia:** *Smoking-(pw4a)-visceral adiposity-(pw30)-*↓*HDL-(pw31)-*↑ *Hypercholesterolemia*.
- **Inflammatory state:** *Smoking-(pw4b)-central nervous system-(pw41)-*↑*TNF-*α*-(pw41)-*↑*Inflammatory state*.
- **Hypercoagulability:** *Smoking-(pw4a)-visceral adiposity-(pw49)-*↑*Fibrinogen-(pw73)-*↑*Hypercoagulability*.

The activation of these pathways and respective CHD hallmarks may explain some of the increased risk of smokers developing severe Covid-19 compared to non-smokers (87).

#### Oral Health

Using Figure 1, we published a detailed analysis of the mechanism by which oral health (in the form of periodontal disease) can influence CHD (27). Important elements relevant to this study are discussed below.

Oral health in the form of periodontal disease is known to increase the risk of CHD by 1.34-fold (88) (Figure 11 and Table 2). Covid-19 patients with periodontitis have a much higher risk of mortality, OR of 8.81 (Figure 11 and Table 2) (89). This value is quite large and could be overestimated. There are several reasons for potential overestimation namely, the small study size (*n* = 568), the data is widely spread (95% CI of 1.00–77.7) and the data is not statistically significant [this statistical insignificance is illustrated on Figure 11 with an (^*^)] (89).

Nevertheless, the increased risk of Covid-19 severity due to periodontitis could partially be explained by the increase in several CHD hallmarks namely, *Inflammatory state, Hypercoagulability and Hypercholesterolemia* (23, 27).

An increased risk of *Hypercoagulability* and *Inflammation* in these patients is through a common periodontitis associated bacteria, porphyromonas gingivalis (p.gingivalis) (127). This bacteria invades endothelial cells which concomitantly increases platelet activity and stimulates proinflammatory mediators/cytokines (CRP, TNF-α, and IL-6) (127).

*Inflammation* can also be increased *via* reactive oxygen species (ROS) which is associated with periodontal disease (23, 27). Subsequently, this also affects oxidized LDL levels pertaining to an increase in the risk for *Hypercholesterolemia* (23, 27).

The associated pathways and respective CHD hallmarks increased by oral health in the form of periodontitis are shown in Figure 4 as the following:

- **Hypercoagulability:** *Oral health-(pw5)-periodontium-(pw23)-*↑*P. gingivalis-(pw43)-*↑*periodontitis-(pw64)-*↑*platelet factors-(pw73)-*↑*Hypercoagulability*.
- **Inflammatory state**: *Oral health-(pw5)-periodontium-(pw23)-*↑*P. gingivalis-(pw43)-*↑*periodontitis-(pw41)-*↑*TNF*α*/IL6-(pw41)-*↑*inflammatory state*. *Oral health-(pw5)-periodontium-(pw23)-*↑*P. gingivalis-(pw43)-*↑*periodontitis-(pw62)-*↑*ROS-(pw85)-*↑*inflammatory state*.
- **Hypercholesterolemia:** *Oral health-(pw5)-periodontium-(pw23)-*↑*P. gingivalis-(pw43)-*↑*periodontitis-(pw65)-*↑*oxLDL-(pw51)-*↑*Hypercholesterolemia*.

The potential increase of these CHD hallmarks due to periodontitis could partially explain the increased risk of Covid-19 severity.

#### Chronic Stress

Chronic stress (definition in section Evaluation of *Health Factors* and *Pharmaceutical Interventions*) is also a common factor linked to an increased risk for CHD, with an OR of 2.17 (90), presented in Figure 11 and Table 2. Covid-19 severity is also increased by chronic stress with a HR of 1.4 (91), see Figure 11.

Chronic stress is known to elevate secretion of glucocorticoids in the form of cortisol. These high cortisol levels due to stress may elevate biomarkers such as blood glucose, TNF-α and insulin resistance (23). These stress related biomarkers are also upregulated in severe Covid-19 patients (23, 75, 76, 128–131).

The respective CHD hallmarks and activated pathways activated by chronic stress are denoted in Figure 4 as:

- **Hypercoagulability:** *Chronic stress-(pw6)-central nervous system-(pw27)-*↑*cortisol-(pw48)-liver-(pw14)-*↑*blood glucose-(pw54)-PI3K:MAPK-(pw69)-*↑*insulin resistance-(pw72)-*↑*platelet factors-(pw73)-*↑*Hypercoagulability*.
- **Hypercholesterolemia:** *Chronic stress-(pw6)-central nervous system-(pw27)-*↑*cortisol-(pw48)-liver-(pw12)-*↑*LDL-(pw33)-*↑*oxLDL-(pw51)-*↑*Hypercholesterolemia*.
- **Hyperglycemia/Hyperinsulinemia:** *Chronic stress-(pw6)-central nervous system-(pw27)-*↑*cortisol-(pw48)-liver-(pw14)-*↑*blood glucose-(pw54)-PI3K:MAPK-(pw69)-*↑*insulin resistance-(pw72)-*↑*Hyperglycemia/Hyperinsulinemia*.
- **Inflammatory state:** *Chronic stress-(pw6)-central nervous system-(pw27)-*↑*cortisol-(pw48)-liver-(pw14)-*↑*blood glucose-(pw54)-PI3K:MAPK-(pw69)-*↑*insulin resistance-(pw70)-*↑*angiotensin II-(pw88)-renin-(pw50)-*↑*TNF*α*-(pw41)-*↑*Inflammatory state*.
- **Hypertension:** *Chronic stress-(pw6)-central nervous system-(pw27)-*↑*cortisol-(pw48)-liver-(pw14)-*↑*blood glucose-(pw54)-PI3K:MAPK-(pw69)-*↑*insulin resistance-(pw70)-*↑*angiotensin II-(pw89)-* ↑*Hypertension*.

Although the Covid-19 study is small (*n* = 535, see Table 2) stress affects all five CHD hallmarks. Future larger clinical studies are expected to emphasize the importance of stress management in patients with Covid-19.

#### Depression

The effect of depression on CHD, using the CHD model in Figure 1, was described in detail in a previous paper (24). A summary of the potential effects of depression on Covid-19 are given in the rest of this section.

Depression increases one's risk for CHD by 1.90-fold (RR) (92), shown in Figure 11 and Table 2. This is also the case for Covid-19, where the odds of developing more severe disease in a person with pre-pandemic depression is 2.68-fold (OR) higher that without depression (93), see Figure 11.

Depression is thought to mediate, among others, over stimulation of the hypothalamic-pituitary-adrenocortical (HPA) axis induced by elevated levels of corticotropin-releasing factor and adrenocorticotropic hormone (23, 24). Chronic dysregulation of the hypothalamic-pituitary-adrenal axis can lead to increased serum levels of cortisol. Similar to chronic stress, elevated cortisol levels can increase the risk of upregulating four CHD hallmarks namely, *Inflammatory state, Hypercholesterolemia, Hypertension* and *Hyperglycemia/Hyperinsulinemia* (23, 24).

In addition to increased cortisol levels, the overstimulation of the hypothalamic-pituitary-adrenal axis may augment sympathoadrenal hyperactivity *via* central regulatory pathways. This results in increased plasma catecholamines (23, 24). An increase of catecholamines can lead to abnormalities in insulin and platelet factors thus also increasing another CHD hallmark namely, *Hypercoagulability* (23, 24).

The respective CHD hallmarks and activated pathways induced by depression are denoted in Figure 4 as the following:

- **Hypercholesterolemia:** *Depression-(pw7b)-central nervous system-(pw27)-*↑*cortisol-(pw48)-liver-(pw12)-*↑*LDL-(pw33)-*↑*oxLDL-(pw51)-*↑*Hypercholesterolemia*.
- **Inflammatory state:** *Depression-(pw7b)-central nervous system-(pw27)-*↑*cortisol-(pw48)-liver-(pw14)-*↑*blood glucose-(pw54)-PI3K:MAPK-(pw69)-*↑*insulin resistance-(pw70)-*↑*angiotensin II-(pw88)-renin-(pw50)-*↑*TNF*α*-(pw41)-*↑*Inflammatory state*.
- **Hypertension:** *Depression-(pw7b)-central nervous system-(pw27)-*↑*cortisol-(pw48)-liver-(pw14)-*↑*blood glucose-(pw54)-PI3K:MAPK-(pw69)-*↑*insulin resistance-(pw70)-*↑*angiotensin II-(pw89)-* ↑*Hypertension*.
- **Hyperglycemia/Hyperinsulinemia:** *Depression-(pw7b)-central nervous system-(pw26)-*↑*catecholamines /* ↓*serotonin /* ↓*BDNF-(pw44)-*↑*insulin resistance-(pw72)-* ↑*Hyperglycemia / Hyperinsulinemia*.
- **Hypercoagulability:** *Depression-(pw7b)-central nervous system-(pw26)-*↑*catecholamines /* ↓*serotonin /* ↓*BDNF-(pw44)-*↑*insulin resistance-(pw72)-*↑*platelet factors-(pw73)-*↑*Hypercoagulability*.

Since depression can upregulate all five CHD hallmarks (23, 24), it may play a more important role in Covid-19 severity than expected.

#### Apnoea

Figure 11 and Table 2 show that obstructive sleep apnoea (OSA) is associated with an increased risk for CHD with a HR of 2.06 (94). Among 15 835 Covid-19 patients, those with OSA have a 2.37-fold (OR) increased odds of developing severe Covid-19 (95).

Similar to depression, the effects of OSA may also include alterations of the hypothalamic-pituitary-adrenal axis and sympathetic nervous activity. This results in changes of catecholamine and cortisol secretion levels, which concomitantly serve to up-regulate two CHD hallmarks namely, *Inflammatory state* and *Hypertension* (23). Subsequently, increased cortisol levels also increases the risk for elevated LDL and platelet factors, which influence the risk for two more CHD hallmarks namely, *Hypercholesterolemia* and *Hypercoagulability* (23).

The respective CHD hallmarks and activated pathways induced by OSA are denoted in Figure 4 as the following:

- **Inflammatory state:** *Apnoea-(pw9)-central nervous system-(pw27)-*↑*cortisol-OSA-(pw42)-hypoxia-(pw61)-*↑*ROS-(pw85)-*↑*Inflammatory state*.
- **Hypertension:** *Apnoea-(pw9)-central nervous system-(pw27)-*↑*cortisol-OSA-(pw42)-*↑*hypoxia-(pw42)-*↑*)-oxia-(pSA-(pus systw70)-*↑*angiotensin II-(pw89)-*↑*Hypertension*.
- **Hypercholesterolemia:** *Apnoea-(pw9)-central nervous system-(pw27)-*↑*cortisol-(pw48)-visceral adiposity-(pw21)-*↑*TNF-*α*/IL-6-(pw56)-liver-(pw12)-*↑*LDL-(pw33)-*↑*oxLDL-(pw51)-*↑*Hypercholesterolemia*.
- **Hypercoagulability:** *Apnoea-(pw9)-central nervous system-(pw27)-*↑*cortisol-(pw47)-*↑*insulin resistance-(pw42)-*↑*platelet factors-(pw73)-*↑*Hypercoagulability*.

The activation of proinflammatory mediators namely, TNF-α, IL-6 and CRP induced by OSA are also elevated in severe Covid-19 patients without OSA (20–22). Therefore, OSA could aggravate these mediators, leading to an increased risk of Covid-19 severity.

#### Insomnia

Insomnia is another *health factor* that increases a person's risk for CHD with a RR of 1.45 (96), see Figure 11 and Table 2. The effect of insomnia on increased Covid-19 severity seems negligible with an OR of 1.09 (97), see Figure 11. Unfortunately the study is small (*n* = 568) and the data are statistically insignificant (95% CI of 0.44–2.71) (97), see Table 2 and Figure 11.

Nevertheless, insomnia affects several pathogenic pathways that may play an important role in Covid-19 severity (23). Insomnia has shown to directly affect the levels of leptin (decreases) and ghrelin (increases), which are important hormones that regulate appetite. This could cause an increase in caloric consumption which, if left untreated, could negatively impact blood glucose levels and insulin sensitivity (23). This would therefore result in an increased risk for *Hyperglycemia/Hyperinsulinemia* (23).

Subsequently, insulin resistance stemming from excessive caloric intake can stimulate proinflammatory mediators and cytokines such as TNF-α, IL-6 and CRP. This could result in a heightened *Inflammatory state*, which is common in severe Covid-19 patients (20–23). Another CHD hallmark upregulated by insulin resistance through the regulation of platelet homeostasis is *Hypercoagulability* (23). Coagulation is also a common risk factor in severe Covid-19 patients (4, 6–9, 23).

The respective CHD hallmarks and activated pathways induced by insomnia are denoted in Figure 4 as the following:

- **Hyperglycemia/Hyperinsulinemia:** *Insomnia-(pw8b)-central nervous system-(pw25)-(pw66)-*↑*ghrelin:leptin-(pw67)-*↑*insulin resistance-(pw72)-liver-(pw14)-*↑*blood glucose-(pw55)-*↑*Hyperglycemia/Hyperinsulinemia*.
- **Inflammatory state:** *Insomnia-(pw8b)-central nervous system-(pw25)-(pw66)-*↑*ghrelin:leptin-(pw67)-*↑*insulin resistance-(pw70)-*↑*angiotensin II-(pw88)-renin-(pw50)-*↑*TNF-*α*, IL-6-(pw41)-*↑*Inflammatory state*.
- **Hypercoagulability:** *Insomnia-(pw8b)-central nervous system-(pw25)-(pw66)-*↑*ghrelin:leptin-(pw67)-*↑*insulin resistance-(pw72)-*↑*platelet factors-(pw73)-*↑*Hypercoagulability*.

Unfortunately, the clinical data on insomnia and its effect on Covid-19 severity are small. Its effect may be underestimated.

#### Moderate Alcohol Use

The mechanism by which moderate alcohol consumption may influence CHD was described in detail in our previous paper (26). Moderate alcohol consumption is accepted to reduce the risk of CHD (23, 26, 98). Table 2 shows a decrease risk (RR) of 0.75 (*n* = 645 087, *N* = 33) (98). This translates to a 1.41-fold decrease in CHD risk (23, 26), illustrated in Figure 11. This decrease in CHD risk may be due to several pathways that decrease the risk for CHD hallmarks.

Moderate alcohol consumption may reduce fibrinogen levels, clotting factors, and platelet aggregation. Downregulation of these biomarkers reduces a state of *Hypercoagulability* (26). In addition, it can also upregulate HDL and downregulate LDL, which decrease *Hypercholesterolemia* (26).

Moreover, moderate alcohol consumption can reduce hepatic gluconeogenesis and concomitantly decrease plasma glucose levels, which decreases the incidence of *Hyperglycemia* and *Hyperinsulinemia* (26). Lastly, it can serve to reduce chronic *Inflammation* through regulation of insulin resistance (26).

These respective CHD hallmarks and pathogenic pathways activated by moderate alcohol consumption (26), are denoted in Figure 4 as:

- **Hypercoagulability:** *Alcohol-(pw1)-Liver-(pw49)-* ↓*fibrinogen/clotting factors-(pw73)-* ↓*Hypercoagulability* and *Alcohol-(pw1)-Liver-(pw49)-*↑*fibrinogen/clotting factors-(pw75)-* ↓*platelet aggregation*.
- **Hypercholesterolemia:** *Alcohol-(pw1)-Liver-(pw10)-*↑*HDL-(pw31)-*↓*Hypercholesterolemia* and *Alcohol-(pw1)-Liver-(pw12)-*↓*LDL-(pw33)- oxLDL-(pw51)-*↓*Hypercholesterolemia*.
- **Hyperglycemia/Hyperinsulinemia:** *Alcohol-(pw1)-Liver-(pw14)-*↓*blood glucose-(pw55)-*↓*Hyperglycemia/Hyperinsulinemia*.
- **Inflammation:** *Alcohol-(pw1)-Liver-(pw14)-*↓*blood glucose-(pw54)-PI3K:MAPK-(pw69)-insulin resistance-(pw70)-Angiotensin II-(pw89)-*↓*Hypertension-(pw100)-*↓*ROS-(pw85)-*↓*Inflammatory state*.

These pathways demonstrate an important role moderate alcohol consumption plays in four of the five CHD hallmarks. The argument whether moderate alcohol consumption before infection decreases or increases Covid-19 severity has not yet been thoroughly explored.

However, the prevailing point of view is that alcohol consumption during Covid-19 could increase Covid-19 severity (132). This is due to alcohol increasing the risk of acute respiratory distress syndrome and admission to intensive care unit in patients with pneumonia (132, 133). These are common risk factors in critical Covid-19 patients (132, 133).

Increased hypercoagulability, Hyperglycemia and inflammation are common in severe Covid-19 patients (4, 6–9, 11, 13, 14). Therefore, the reduction of these CHD hallmarks by moderate alcohol consumption before infection of SARS-CoV-2 could be advantageous. It seems to create a better vascular “baseline” and could thus potentially reduce the risk of developing severe Covid-19 complications. These effects should however be studied in well-designed clinical trials.

#### Food Intake (High Glycemic Diets)

We have previously explained, with reference to Figure 1, how high glycemic diets (HGDs) affect CHD (25). Only a summary of the elements relevant to Covid-19 are given below.

A high glycemic diet (HGD) increases the risk for CHD with a RR of 1.36 (99), see Figure 11 and Table 2. These diets could play an important role in Covid-19 severity through regulation of all five CHD hallmarks (23, 25).

HGDs influences glycemic control by raising blood glucose levels *via* carbohydrate consumption. This may result in *Hyperglycemia* (23, 25). *Hyperglycemia* resulting from HGDs can increase the risk of insulin resistance by upregulating the Phosphatidylinositol 3-kinase : Mitogen-activated protein kinase (PI3K:MAPK) ratio (23, 25). Subsequently, an increased insulin resistance has been associated with increased levels of platelet factors that upregulate the potential for *Hypercoagulation* (23, 25).

Excessive intake of HGDs can result in increased adipose tissue, which enhances pro-inflammatory mediators such as CRP and TNF-α (23, 25). These mediators are, among others, important to consider since they are upregulated in critical Covid-19 patients (129, 130, 134–140). Macrophages, residing in adipose tissue, are also one of the most active secretory cells in the body that mediate activities of adipocytes and release a vast array of inflammatory mediators (23, 25). This increases the risk for an *Inflammatory state*.

Moreover, excessive intake of HGDs can also increase visceral fat build up and reduce clearance of triglycerides, which leads to increased LDL and decreased HDL levels (23, 25). This constitutes to a potential risk of *Hypercholesterolemia* (23, 25). Consequently, HGDs pertaining to visceral fat build up also increases the risk of *Hypertension*. This happens through build-up of excess adipose tissue, which increases the expression of angiotensinogen thus leading to activation of the renin-angiotensin system (23, 25).

These respective CHD hallmarks and pathogenic pathways activated by HGD are denoted in Figure 4 as the following:

- **Hyperglycemia:** *Food-(pw2)-gastro-intestines-(pw17)-*↑*blood glucose-(pw55)-*↑*Hyperglycemia*.
- **Hypercoagulability:** *Food-(pw2)-gastro-intestines-(pw17)-*↑*blood glucose-(pw54)-*↑*PI3K:MAPK-(pw69)-*↑*insulin resistance-(pw72)-*↑*platelet factors-(pw73)-*↑*Hypercoagulability*.
- **Inflammatory state:** *Food-(pw2)-gastro-intestines-(pw15)-plasma lipids-(pw34)-liver-(pw13)-TMAO/NLRP3-(pw52)-macrophage-(pw77)-*↑*Inflammatory state*.
- **Hypercholesterolemia:** *Food-(pw2)-gastro-intestines-(pw15)-plasma lipids-(pw34)-liver-(pw12)-*↑*LDL-(pw33)-oxLDL-(pw51)-*↑*Hypercholesterolemia*.
- **Hypertension:** *Food-(pw2)-gastro-intestines-(pw14)-blood glucose-(pw54)-*↑*angiotensin II-(pw89)-*↑*Hypertension*.

These pathways demonstrate the detrimental effect HGDs may have on an individual's “baseline” vascular system before infection from SARS-CoV-2. It could potentially increase the risk of developing severe Covid-19 complications.

### Effects of Different CHD *Pharmaceutical Interventions* on Covid-19 Severity

The *integrated CHD/Covid-19 model* shows that similar outcomes for different *health factors* are seen in CHD and Covid-19. The next question is: Since we know that various *pharmaceutical interventions* decreases one's risk for CHD, will they also work for Covid-19? If they do then this will further show validity of the proposed *integrated CHD/Covid-19 model*.

The *pharmaceutical interventions* are shown in Figure 4 as blue boxes, where blunted blue arrows *(*
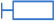
*)*denote antagonize or inhibit and pointed blue arrows *(*
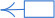
*)* denote up-regulate or facilitate. The question is whether these pharmaceuticals would also decrease one's risk for severe Covid-19. This was investigated, despite the limited clinical data available for Covid-19. The data were extracted from literature and are summarized in Table 2.

No risk value was available for antidepressants' effect on Covid-19 severity. However, its effect on Covid-19 severity is still discussed in this section as depression was shown to increase the odds of developing severe Covid-19 complications by 2.68 (Section Effects of Different CHD *Pharmaceutical Interventions* on Covid-19 Severity, Table 2). It is thus hypothesized that certain anti-depressants should have an important influence on Covid-19 severity.

In the rest of this section we will discuss, in more detail, the effects each *pharmaceutical intervention* has on the CHD hallmarks, and how this could affect Covid-19 severity.

#### Statins

**Figure 12:**
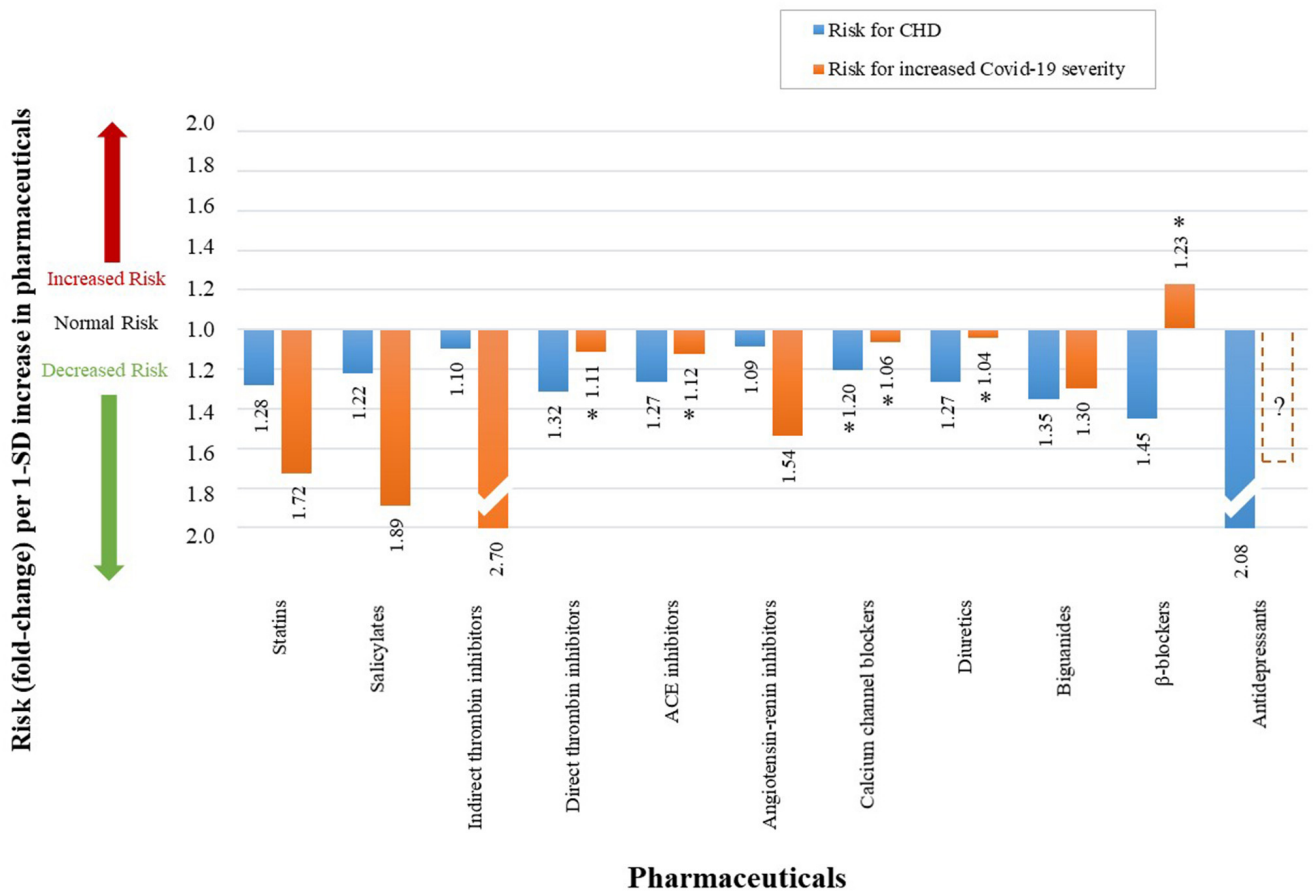
The qualitative effect that different pharmaceuticals have on CHD risk and Covid-19 severity. (An accurate quantitative comparison is not possible, mostly due to differences in study design and size).

The use of statins decreases the risk of CHD with a RR of 0.78 (100). This translates to a 1.28-fold decrease in CHD risk (23), illustrated in Table 2 and Figure 12. Statins also decrease Covid-19 severity, with a HR of 0.58 (*n* = 13 981) (101) (Table 2). This translates to a decrease of Covid-19 severity by 1.72-fold as shown in Figure 12. We evaluated the effects statins has on all of the CHD hallmarks, which may partially explain the large reduction in Covid-19 severity with the use of statins.

Firstly, statins cholesterol lowering effect inhibits the following pathways in Figure 4: (pw11) and (pw12). Besides these cholesterol lowering effects, it also has an anti-inflammatory effect (23, 101). The anti-inflammatory biomarkers and pathways on which inhibition is observed are denoted in Figure 4 as: NFκβ, ROS and (pw21), (pw57), (pw74).

In addition to their beneficial effects on cholesterol and inflammation, statins also have antihypertensive effects by reducing systolic, diastolic and mean arterial blood pressure (141). The hypertensive pathways on which its actions are observed are denoted in Figure 4 as (pw88) and (pw89).

#### Salicylates

Salicylates such as aspirin is a common anti-inflammatory (142) and anti-thrombotic (143) medication that decreases the risk for CHD with a RR of 0.82 (102), see Table 2. This translates to a 1.22-fold decrease in CHD risk (23), illustrated in Figure 12. Its use in Covid-19 patients also showed a decrease in severity with HR of 0.53 (58). This is shown in Figure 12 as a 1.89-fold decrease in Covid-19 severity (58).

This reduction in risk could be expected because of the detrimental effect of inflammation and coagulation seen in most severe Covid-19 patients (4, 6–9, 20–22). The pathways on which aspirin's actions are observed are denoted in Figure 4 as (pw73) and (pw74).

#### Indirect Thrombin Inhibitors

Indirect thrombin inhibitors such as heparin is used as an anticoagulant, which decreases the odds of CHD with OR 0.91 (103), see Table 2. This translates to a 1.10-fold decrease in CHD risk (23) as shown in Figure 12. Since many severe cases of Covid-19 present venous thromboembolisms and microthrombi (4, 6–9), indirect thrombin inhibitors should be of benefit to such cases.

Heparin was thus expected to reduce these thrombi and reduce Covid-19 severity. A small retrospective analysis (*n* = 449) investigated heparin's effect in Covid-19 patients (104). The study found an OR of 0.37 in Covid-19 mortality (104), see Table 2. This is illustrated in Figure 12 as a 2.7-fold decrease in odds of developing severe Covid-19 (104). The coagulation pathway on which heparin's action is observed is shown in Figure 4 as (pw73).

Heparin also seems to have an anti-inflammatory effect (144), which is presented in Figure 4 as pathway (pw74). This effect is however only seen at much higher concentrations which could increase the risk of bleeding (144). Therefore, heparin's anti-thrombotic effect would predominantly be the reason for lower Covid-19 severity.

#### Direct Thrombin Inhibitors

Direct thrombin inhibitors have shown to decrease the risk of CHD with HR of 0.76 (105), see Table 2. This translates to a 1.32-fold decrease in CHD risk (23), illustrated in Figure 12. These pharmaceuticals' actions are also observed on the coagulation pathway (pw74) (23), see Figure 4.

For Covid-19, direct thrombin inhibitors are shown to slightly reduce the risk of developing severe disease with a HR of 0.90 (106), see Table 2. This translates to a 1.11-fold reduction in risk (106), illustrated in Figure 12. However, as shown in Figure 12 by an (^*^), this value is not statistically significant with the 95% CI of 0.71–1.15 presented in Table 2.

#### Antihypertensive Pharmaceuticals

The antihypertensive *pharmaceutical interventions* in Figure 4 are: ACE inhibitors, angiotensin-renin inhibitors, β-blockers, calcium channel blockers and diuretics. The pathways on which their actions are observed are shown in Figure 4 as (pw88), (pw89), and (pw50) (23).

The respective reduction of CHD risks for each pharmaceutical (23, 107, 109, 111, 113) is given in Figure 12 and Table 2 as the following:

- Angiotensin-renin inhibitors: 1.09 (OR of 0.92)
- Calcium channel blockers: 1.20 (OR of 0.83)
- ACE inhibitors: 1.27 (OR of 0.79)
- β-blockers: 1.46 (RR of 0.69)
- Diuretics: 1.27 (RR of 0.79)

The reduction in CHD risk is small for angiotensin-renin inhibitors with an OR close to one (0.92) (109). However, angiotensin-renin inhibitors seem to be more beneficial for Covid-19 severity with a reduction in 1.54-fold (110), see Figure 12.

The risk data for calcium channel blockers (OR of 0.94) (112), ACE inhibitors (HR of 0.89) (108) and diuretics (OR of 0.96) (112) are currently not associated with Covid-19 severity. All risk values are close to one and the respective 95% CI's all show statistically insignificance (containing 1.0), see Table 2. This statistically insignificance is illustrated in Figure 12 with an (^*^).

The most interesting of the antihypertensive *pharmaceutical interventions* is β-blockers, which reduced the risk of CHD (RR of 0.69). However, its use increases ones odds of developing severe Covid-19 (OR of 1.23) (112), see Figure 12. The reason for this is unclear and further studies are warranted to investigate the mechanism of action involved. However, one explanation for this difference could be that the data is insignificant for Covid-19, with a 95% CI of 0.74-2.04 (112), see Table 2.

#### Biguanides

Biguanides such as metformin has been used for many decades to treat type 2 diabetes and its use decreases the odds of developing CHD, with a OR of 0.74 (114), see Table 2. This translates to a 1.35-fold decrease in CHD risk (23), illustrated in Figure 12. Metformin's inhibition is observed on pathway (pw14) (23), see Figure 4.

Elevated blood glucose levels at admission is an independent predictor of Covid-19 severity irrespective of diabetes (75). Therefore, glucose lowering agents are expected to reduce Covid-19 mortality. This is indeed the case since a large observational cohort study of type-2 diabetics (*n* = 1 800 005) showed that the use of metformin decreased Covid-19-related mortality by 1.30-fold (HR of 0.77) (115), see Figure 12 and Table 2.

#### Antidepressants (SSRIs)

We have done a detailed study of the mechanisms by which SSRI antidepressants may reduce CHD risk (24). We showed that SSRIs can influence most of the CHD hallmarks (24). A summary, relevant to Covid-19, is given below.

Selective serotonin uptake inhibitors (SSRIs) such as sertraline has shown to decrease the risk of CHD, with a HR of 0.48 (116), see Table 2. This translates to a 2.08-fold decrease in CHD risk (23), illustrated in Figure 12. Sertraline's actions are observed on the anti-inflammatory pathway (pw94), as shown in Figure 4.

A similar SSRI antidepressant, fluvoxamine's effect on Covid-19 severity is currently being investigated in a clinical trial (NCT04727424). This study was initiated by results from a small double-blind, randomized clinical trial of 152 Covid-19 positive patients treated with fluvoxamine (117). The outcomes of this study showed that patients treated with fluvoxamine, compared with a placebo, had a lower likelihood of clinical deterioration (0% vs. 8.3%) (117).

The study did not report any risk data. For this reason, the data could not be added to Figure 12 and Table 2. Nevertheless, since a therapeutic effect is seen in the small Covid-19 study, a dotted bar was added to Figure 12. We hypothesize, based on our previous studies (24), that most SSRIs will be beneficial.

## Discussion and Future Research

The aim of this paper was to use a systems approach to explore the mechanisms between severe Covid-19 and its cardiovascular complications, as requested by *Frontiers*. The resulting *integrated CHD/Covid-19 model* may provide insight into the various research questions, some also requested by *Frontiers*.

### Why Do Some Patients With Severe Covid-19 Experience Sudden Death?

Although aspects of this has been proposed elsewhere (19, 22, 138, 145–148), here its integrated mechanism is systematically and visually shown with the relevant pathogenetic pathways with reference to CHD. This model further elucidates other underlying pathogenesis that may influence this *death spiral* before infection of SARS-CoV-2.

The *death spiral* was summarized as follows: Increased inflammation at the lungs causes EC injury, which can result in vascular leakage and/or activation of the coagulation cascade at the lungs, thereby causing hypoxia which can further increase inflammation, creating two closed positive feedback loops and causing severe Covid-19 through a *death spiral* (Figure 5).

### How Do CHD Comorbidities Influence This *Death Spiral*?

It is widely accepted that patients with pre-existing CHD comorbidities (thus a poor initial vascular “baseline”) have a high risk of developing severe Covid-19 (11–14). The detailed mechanisms of how these comorbidities may influence the *death spiral* was not fully integrated before.

This question was answered in this paper by visually (Figures 8–10) detailing the mechanisms of how three CHD comorbidities namely *Hypercholesterolemia, Hyperglycemia/Hyperinsulinemia* and *Hypertension* can fuel the *death spiral*.

### How Can an Individual Reduce the Risk of Developing Severe Covid-19 From a Cardiovascular Point of View?

In literature different *health factors* (85, 87, 89, 91, 93, 95, 97) and CHD related *pharmaceuticals* (58, 101, 104, 106, 108, 110, 112, 115) present either a reduction or aggravation of Covid-19 severity. In this paper we provide the pathogenesis detailing the effect these *health factors* and *pharmaceuticals* may have on this *death spiral*, especially for those with an increased risk for CHD.

We have shown that severe Covid-19 and CHD have similarities in underlying pathogenesis. Therefore, following a lifestyle that would decrease one's risk for CHD before onset of Covid-19 should also decrease the chances of developing severe Covid-19.

The remaining two research questions [(4) and (5)] have partially been answered by the model but future research is still needed. These are discussed in more detail in the following two Sections.

### How Can Computational Analysis Help to Assess the Risk of Severity in Covid-19 in Cardiovascular Disease?

One of the research questions posed by *Frontiers* in their request for papers was the following: “*How can computational analysis help to assess the risk of COVID-19 in cardiovascular disease?”* We speculate that to achieve such an outcome, at least the following must be done:

**Step 1:** Development of a fully integrated network model for the disease, accounting for all effects including cross linking.

**Step 2:** Characterization of each interaction (typically at the nodes of the network in Step 1) is needed to solve the network.

A first attempt at Step 1 for severe Covid-19 in cardiovascular disease was done in this paper (Figure 4). The next step is characterization of the network using the following equation:

(1) $$\begin{array}{r} Out_{1\to n}=\;f_{1\to n}In_{1\to n} \end{array}$$

where *In*_1 → *n*_ are the inputs (1 to *n*) to a node and *out*_1 → *n*_ are the outputs (1 to *n*) from the node and *f* (1 to *n*) are the resulting transfer functions. The inputs and outputs are typically measured. More detail of this process is given in (23).

Using this process we have developed, over the past four decades, simulation software to solve complex engineering networks e.g., in deep mines and industrial complexes (149). Fortunately, in engineering it is easy to develop transfer functions (Equation 1) as it is relatively easy to do the required measurements. The challenge for medical networks is the measurements of all the relevant pathways in Figure 4.

**Figure 13:**
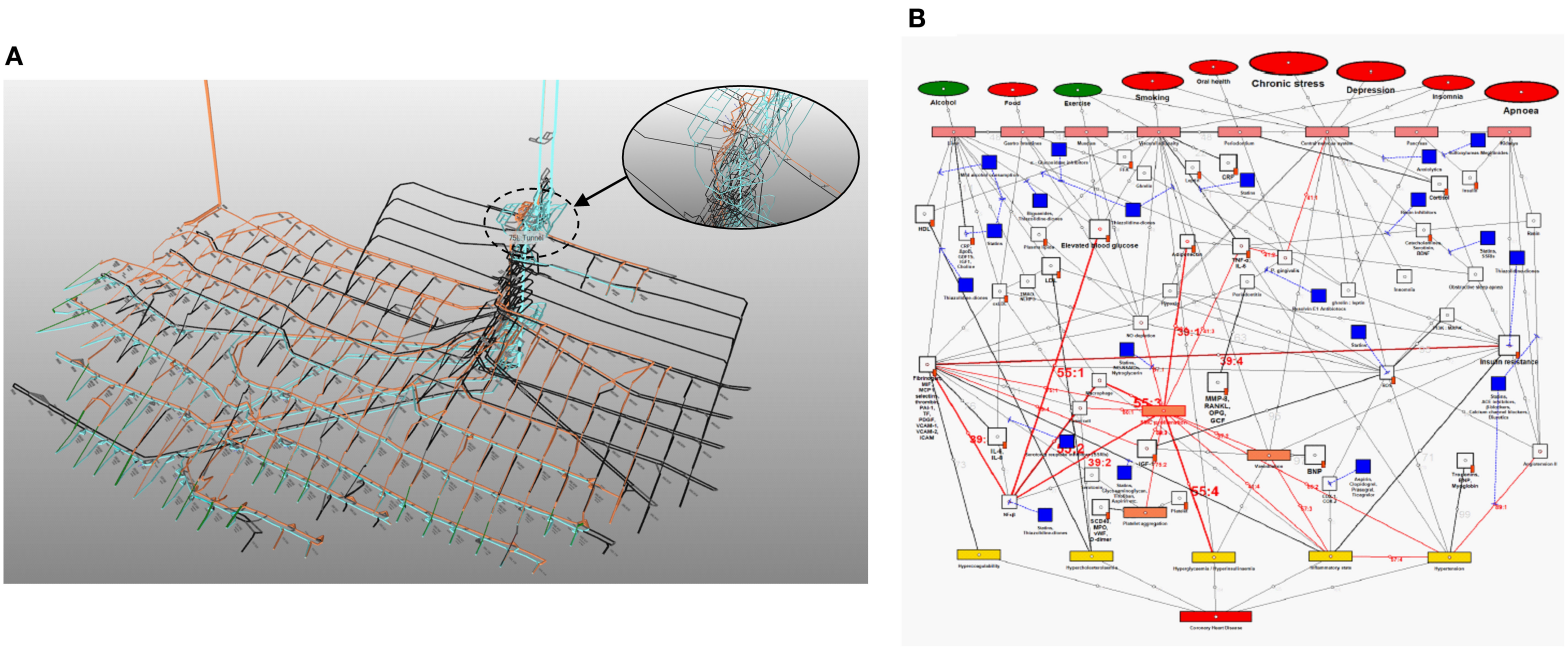
Schematics of typical simulation networks for **(A)** engineering and **(B)** CHD (23). **(A)** provides a small section of computer model of a relatively complex deep mine. **(B)** shows the initial computer model (23) developed from the existing CHD model in Figure 1 (23) using the simulation software developed for **(A)**. The CHD computer model includes all the known interactions for *health factors*, measured elements (salient biomarkers) and controls (*pharmaceutical interventions)*. Remember it is the measured biomarkers that individualize a patient.

A typical deep level mine simulation model (A) and a CHD simulation model (B) proposed in (23) are shown in Figure 13. The following has to be investigated in the future: if all the biomarkers can be measured for the proposed *integrated CHD/Covid-19 model*, will it be possible to individualize the network (Figure 4), thereby making it patient specific? This could be similar to us individualizing our engineering simulations to a specific mine.

The question is: can the risk for Covid-19 severity in CHD then be established for any specific individual by inputting the measured biomarkers of that person into the simulation model based on Figure 4? We have already attempted Step 1. However, for Step 2 much work and data are still needed.

Fortunately, there are numerous clinical trials currently underway that focus on treatments for Covid-19 with respect to the CHD hallmarks, namely the following:

(A) *Hypercoagulation* (122 trials)
(B) *Hypercholesterolemia* (23 trials)
(C) *Hyperglycemia /Hyperinsulinemia (Diabetes)* (84 trials)
(D) *Inflammation* (265 trials)
(E) *Hypertension* (68 trials)

The clinical trial numbers and respective treatments are available as Supplementary Data. The total number of registered clinical trials on CHD hallmarks effect on Covid-19 are 562. This also includes 94 duplicate studies that focus on more than one CHD hallmark.

If we can successfully develop a simulation model, could the best control strategy (pharmaceuticals) be calculated for each individual Covid-19 or CHD patient? This would be similar to identifying the optimum control strategies, which we routinely calculate in engineering, for each individual mine or industrial complex.

However, we acknowledge that there are many assumptions and restrictions relevant to a CHD/Covid-19 computational analysis, which is completely speculative at present. For example, research is needed to investigate how individualized predictions will be feasible. The full details on the research question of computational analysis will be the purpose of future papers.

### Are There Other Opportunities in Cardiovascular Disease That Can Be Derived From This Paper and the Covid-19 Crisis?

We have shown in this paper that the late-stage consequences of severe Covid-19 is often accelerated cardiovascular disease. We have also shown that most *pharmaceutical interventions* which mediate CHD also mediate the effects of Covid-19.

The question arises if the reverse is true. Are there any reported *pharmaceutical interventions* that reduced Covid-19 severity which could potentially be of value for vascular disease? This is an important question as approximately five times more people died during the past year from cardiovascular disease than from Covid-19.

Such a repurposed drug should preferably treat most of the hallmarks of cardiovascular disease. We investigated such a drug namely, ivermectin (150–155). Although ivermectin use is still controversial as a drug against Covid-19, studies over nearly three decades before Covid-19, has shown to reduce four of the five hallmarks of cardiovascular disease. These results and the publication dates are given below:

(A) *Hypercoagulability:* (1992) by increasing prothrombin time in 6.7% (ivermectin group) vs. 1.4% (control group) of participants *in vivo* (humans) (156).
(B) *Hypercholesterolemia:* cholesterol (2013) decreased by 1.5-fold *in vivo* (mice) (157).
(C) *Hyperglycemia:* fasting blood glucose (2013) decreased by 1.4-fold *in vivo* (mice) (157).
(D) *Hyperinsulinemia:* fasting insulin (2013) decreased by 2.0-fold *in vivo* (mice) (157).
(E) *Inflammation:* (2004) decreased IL-1β and TNF-α by 1.27-fold *in vitro* (158).

Except for the CHD hallmark *Inflammation* (159) the focus of ivermectin's proposed mechanism of action (MOA) for Covid-19 is currently on its anti-viral effect (150–153). However, if ivermectin really shows promise for Covid-19 treatment, could the full vascular MOA for ivermectin be as important or even more important than its anti-viral effects?

This research question can only be answered fully by clinical trials, which measure the relevant vascular biomarkers for each CHD hallmark before and after ivermectin use. Side effects of chronic use such as mild elevation of serum aminotransferases should also be investigated (160).

The MOA of ivermectin for prevention of Covid-19, reportedly seen in small studies (161), is also not clear to the authors. Why would the anti-viral MOA of ivermectin have a preventable effect if the patient has not been infected yet?

If ivermectin really helps for prevention of Covid-19, could it rather help create a healthier vascular system (“baseline”) before the virus strikes, especially in vascular compromised individuals, rather than only help *via* its proposed anti-viral effect? Therefore, could ivermectin's effect on the vascular system during severe Covid-19 be its most important MOA?

If well-designed clinical trials show that ivermectin could be a potential cardiovascular drug, could it be an ideal, inexpensive, drug for low- and middle-income countries where a high percentage of global cardiovascular related deaths occur (162)?

### Other Research Questions Emanating From This Study

Other research questions that should be investigated in future research are the following:

1. Why would β -blockers have an opposite effect on Covid-19 severity than on CHD?
2. There exists an anomaly between statin's cholesterol lowering effect and low cholesterol levels seen in end-stage Covid-19. How can this drug help decrease Covid-19 severity while it further decreases cholesterol? Can statin's anti-inflammatory effect be so large that it overrides its cholesterol lowering effect? Would it then be better to drop statins and rather only use anti-inflammatory medication? Or does it depend on the stage of the disease, beneficial at first but not in the end stage?
3. Does a high correlation of most CHD related *pharmaceutical interventions* and Covid-19 mean that other CHD pharmaceuticals not investigated in detail for Covid-19 could also help reduce Covid-19 severity?
4. Are there pathways shown in the proposed model (Figure 4) that do not have pharmaceuticals to regulate them? Could this be the focus of new drug discovery for Covid-19 and cardiovascular disease?
5. Could the model be extended to include cerebrovascular disease and other cardiac diseases such as heart failure, valvular heart disease and peripheral artery disease?

## Conclusion

Covid-19 data show that disease severity mostly occurs in patients with pre-existing cardiovascular comorbidities i.e., in patients with poor initial vascular “baselines.” *Frontiers* therefore requested papers on how a systems approach can explore the mechanisms of cardiovascular complications in Covid-19.

This study attempted to fulfill this request by integrating pathways for severe Covid-19 into an existing coronary heart disease (CHD) model. The resulting *integrated CHD/Covid-19 model*, depicted in Figure 4, gives insights into the following issues, some also raised in the *Frontiers* request for research:

- The integrated CHD/Covid-19 pathogenesis of the *death spiral* seen in some critical Covid-19 patients.
- The comprehensive mechanisms of how underlying CHD comorbidities namely, *Hyperglycemia/Hyperinsulinemia, Hypercholesterolemia* and/or *Hypertension* may fuel the *death spiral*.
- The detailed pathogeneses of different *health factors*, which effect CHD risk and Covid-19 severity.
- The mechanisms of how chronic CHD *pharmaceutical interventions* may influence Covid-19 severity.
- The proposed model shows many pathways that currently do not have pharmaceuticals which influence them. This information can be the focus of future drug discovery.
- The proposed model can be further developed as a computational tool not only for Covid-19 application but also for cardiovascular disease.
- Insights into the hallmarks of CHD, shown in the *integrated CHD/Covid-19 model*, also led to various research questions that can form the basis for future research. This includes potential repurposing of an existing drug for cardiovascular disease.

Although the details in this study are complex the message is simple. Studies such as this one not only highlight the value of a cardiovascular healthy lifestyle in general but also specifically for Covid-19. With the sharp focus on Covid-19 we hope that this “healthy living” message will be intensified, thus help to reduce cardiovascular deaths, the prime killer of man.

## Data Availability Statement

The original contributions presented in the study are included in the article/Supplementary Material, further inquiries can be directed to the corresponding author/s.

## Author Contributions

AM developed the first draft (of more than 30) of the manuscript and compiled and analyzed the Covid-19 risk factor data. EM was the principal investigator. During level 5 Lockdown in May 2020 he initiated the original ideas and research including the *death spiral* and potential use of ivermectin for CHD based on MM's Ph.D. AM and AG developed the integrated CHD/Covid-19 model from literature. MM developed the CHD-based model and provided expert opinion for the integration of the model with Covid-19. All authors have assisted in revisions and have approved the final manuscript.

## Funding

The research was funded by EM and HumanSim (Pty) Ltd.

## Conflict of Interest

This study received funding from HumanSim (Pty) Ltd. The funder was not involved in the study design, collection, analysis,interpretation of data, the writing of this article or the decision to submit it for publication. The authors declare that the research was conducted in the absence of any commercial or financial relationships that could be construed as a potential conflict of interest.

## Publisher's Note

All claims expressed in this article are solely those of the author and do not necessarily represent those of their affiliated organizations, or those of the publisher, the editors and the reviewers. Any product that may be evaluated in this article, or claim that may be made by its manufacturer, is not guaranteed or endorsed by the publisher.

## Nomenclature

ACE: Angiotensin-converting-enzyme
β-blocker: Beta-adrenergic antagonists
BDNF: Brain-derived neurotrophic factor
BNP: B-type natriuretic peptide
Covid-19: Coronavirus disease of 2019
COX: Cyclooxygenase
CRP: C-reactive protein
CHD: Coronary heart disease
D-dimer: Fibrin degradation product D
EC: Endothelial cell
FFA: Free fatty acids
GCF: Gingival crevicular fluid
GM-CSF: Granulocyte-Macrophage Colony-Stimulating Factor
HbA1c: Glycated hemoglobin A1c
HDL: High-density lipoprotein
HGD: High Glycemic Diet
HR: Hazard Ratio
Hs: Homocysteine
ICAM: Intracellular adhesion molecule
IGF-1: Insulin-like growth factor-1
IL: Interleukin
LDL: Low-density lipoprotein
MAPK: Mitogen-activated protein kinase
MCP: Monocyte chemoattractant protein
MIF: Macrophage migration inhibitory factor
MMP: Matrix metalloproteinase
MOA: Mechanism Of Action
MPO: Myeloperoxidase
NFκβ: Nuclear factor-kappa-beta
NLRP3: Nod-like receptor family pyrin domain containing 3
NO: Nitric oxide NO-NSAID, Nitric oxide-non-steroidal anti-inflammatory drug
OPG: Osteoprotegerin
OR: Odds Ratio
oxLDL: Oxidized LDL
PAI: Plasminogen activator inhibitor
PDGF: Platelet-derived growth factor
P. gingivalis: Porphyromonas gingivalis
PI3K: Phosphatidylinositol 3-kinase
RANKL: Receptor activator of nuclear factor kappa-beta ligand
ROS: Reactive oxygen species
RR: Relative Risk
SARS-CoV-2: Severe acute respiratory syndrome coronavirus 2
SCD-40: Recombinant human sCD40 ligand
SMC: Smooth muscle cell
SSRI: Serotonin reuptake inhibitors
TF: Tissue factor
TMAO: Oxidation product of trimethylamine
TNF-α: Tumor necrosis factor-alpha
VCAM: Vascular cell adhesion molecule
vWF: von Willebrand factor.
